# Redistribution of Adhesive Forces through Src/FAK Drives Contact Inhibition of Locomotion in Neural Crest

**DOI:** 10.1016/j.devcel.2018.05.003

**Published:** 2018-06-04

**Authors:** Alice Roycroft, András Szabó, Isabel Bahm, Liam Daly, Guillaume Charras, Maddy Parsons, Roberto Mayor

**Affiliations:** 1 Department of Cell and Developmental Biology, University College London, Gower Street, London WC1E 6BT, UK; 2 Randall Division of Cell and Molecular Biophysics, Kings College London, London SE11UL, UK; 3 London Centre for Nanotechnology, UCL, London WC1H 0AH, UK; 4 Institute for the Physics of Living Systems, UCL, London WC1E 6BT, UK

**Keywords:** neural crest, collective cell migration, traction forces, intercellular tension, N-cadherin, FAK, Src, focal adhesion, focal contacts, extracellular matrix

## Abstract

Contact inhibition of locomotion is defined as the behavior of cells to cease migrating in their former direction after colliding with another cell. It has been implicated in multiple developmental processes and its absence has been linked to cancer invasion. Cellular forces are thought to govern this process; however, the exact role of traction through cell-matrix adhesions and tension through cell-cell adhesions during contact inhibition of locomotion remains unknown. Here we use neural crest cells to address this and show that cell-matrix adhesions are rapidly disassembled at the contact between two cells upon collision. This disassembly is dependent upon the formation of N-cadherin-based cell-cell adhesions and driven by Src and FAK activity. We demonstrate that the loss of cell-matrix adhesions near the contact leads to a buildup of tension across the cell-cell contact, a step that is essential to drive cell-cell separation after collision.

## Highlights

• Focal adhesions disassemble at cell-cell contacts in contact inhibition of locomotion
• FA disassembly at the cell contact during CIL requires N-cadherin/Src/FAK signaling
• Cell separation during CIL involves a buildup of tension across the cell contact

## Graphical Abstract

**Figure:**
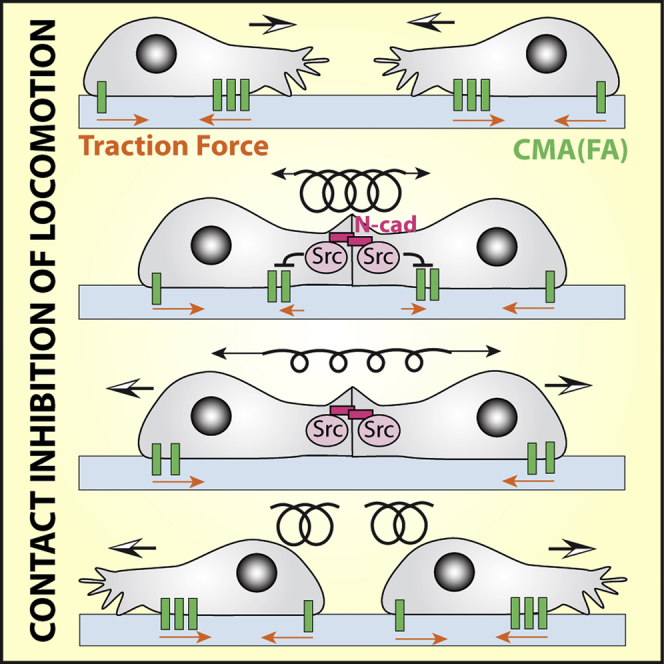

## Teaser

Contact inhibition of locomotion leads to cell separation when they collide during migration. Roycroft et al. examine the role of mechanical force in cell separation and demonstrate that immediately after collision, cell-matrix adhesions are disassembled, leading to a tension buildup across cell-cell contacts that is essential for cell separation.

## Introduction

**Figure 1:**
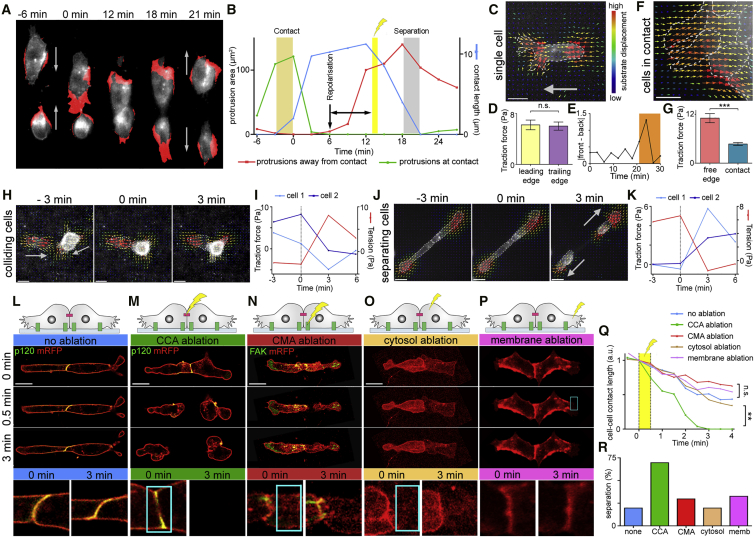
Redistribution of Forces during CIL (A) Membrane GFP-labeled NC cells undergoing CIL. Red indicates the protrusion extension (Figure S1E). (B) Dynamic behaviors of the protrusions toward the contact (green), away from the contact (red), and the length of the cell-cell contact (blue). Beige bar indicates the time when cells collided. Gray bar indicates the time when cells separated. Yellow bar indicates when laser ablation was carried out. (C, F, H, and J) TFM showing substrate displacement vectors. Cells labeled with membrane RFP are outlined in gray. (D and G) Average traction at leading edge/free edge (D) and trailing edge/contact (G). n = 17 for single cells and n = 27 for cells in contact. n.s., not significant. (E) Imbalance of traction at front and rear of a single cell (Pa); orange block indicates when the cell starts to migrate. (I and K) Traction at contact pointing away from the contact over time in colliding/separating cells. 0 min = first frame of contact for (I), and last frame before separation for (K). Tension across the contact is shown in red. (L–P) Schematic of cells and frames from movies as indicated. Cells were injected to express membrane RFP (red) and either p120-catenin-GFP to label CCAs (green in L and M) or GFP- FAK to label cell-matrix adhesions (green in N). Bottom row: zoom of cell-cell contact. Ablation area marked with a cyan box. To maintain the same orientation and scale, images in N–P were rotated, zoomed, and the background was filled with black. (Q) Median value of the length of the cell-cell contact relative to length at the start of laser ablation (0 min). Yellow area shows time of laser ablation. n = 10 for no ablation, CCA ablation, and CMA ablation; n = 8 for cytosol ablation; n = 3 for free membrane ablation. (R) Percentage of cells that separate within 3 min after ablation. Scale bars, 20 μm. Line graphs show medians. Bar graphs show means, errors ± SEM. ^∗∗∗^p ≤ 0.001, ^∗∗^p ≤ 0.01. (D and G) Mann-Whitney test, (K) Kruskal-Wallis test. See also Figure S1.

Contact inhibition of locomotion (CIL) is a migratory phenomenon where cells repolarize and migrate away from each other after coming into contact (Figures 1A and S1A). It was first observed in the 1920s between hemocytes from horseshoe crabs (Loeb, 1921). Abercrombie and Heaysman, 1953, Abercrombie and Heaysman, 1954 further characterized CIL between chick heart fibroblasts in the 1950s. Since then CIL has been shown as the driving force behind many different aspects of development, including the directional migration of *Xenopus* and zebrafish neural crest (NC) (Carmona-Fontaine et al., 2008, Theveneau et al., 2010), the patterning of *Drosophila* hemocytes (Davis et al., 2012), and the directed distribution of mouse Cajal-Retzius neurons throughout the cortex (Villar-Cerviño et al., 2013), and it is also likely to play a role in border cell migration in *Drosophila* (Cai et al., 2014). Further to its role in development, CIL has also been implicated in cancer where the loss of CIL toward healthy tissue can result in metastasis (Abercrombie et al., 1957, Astin et al., 2010). CIL is a multi-step process driven by a variety of different mechanisms and components (Roycroft and Mayor, 2016; Stramer and Mayor, 2017; Mayor and Etienne-Manneville, 2016, Desai et al., 2013, Lin et al., 2015), including elements of the Wnt/PCP pathway (Carmona-Fontaine et al., 2008, Matthews et al., 2008, Theveneau et al., 2013), cadherins (Bahm et al., 2017, Becker et al., 2013, Huttenlocher et al., 1998, Scarpa et al., 2015, Theveneau et al., 2010), ephrins (Batson et al., 2013, Batson et al., 2014, Tanaka et al., 2012, Villar-Cerviño et al., 2013), small GTPases (Anear and Parish, 2012, Kadir et al., 2011, Matthews et al., 2008, Scarpa et al., 2015, Theveneau et al., 2010), and cytoskeleton rearrangements (Davis et al., 2015, Kadir et al., 2011, Moore et al., 2013, Roycroft and Mayor, 2015, Stramer et al., 2010).

Besides the above molecular components, a role for physical forces exerted by the cells during CIL has been discussed (Abercrombie and Ambrose, 1958, Abercrombie and Dunn, 1975, Coburn et al., 2016, Davis et al., 2015, Harris, 1973, Heaysman and Pegrum, 1973, Roycroft and Mayor, 2015, Roycroft and Mayor, 2016, Zimmermann et al., 2016). Cells generate traction forces on the substrate through cell-matrix adhesions (CMAs), which are transmembrane complexes that crosslink the intracellular cytoskeleton to the extracellular matrix (ECM) via integrins and adapter proteins, resulting in force transmission, signaling, and cytoskeletal rearrangements (Ananthakrishnan and Ehrlicher, 2007, Case and Waterman, 2015, Sastry and Burridge, 2000). When, in the 1970s, Harris (1973) first investigated the behavior of adhesion to the substrate during CIL, he observed a loss of cell-substrate adhesion near the contact and speculated that this led to a transfer of tension from the cell-substrate to the cell-cell contact and that this transfer of tension was sufficient to break the adhesions holding the colliding cells together. Abercrombie and Dunn (1975), however, argued that cell-substrate adhesions persisted near the contact and that an alternative mechanism for tension buildup across the cell-cell contact was required. How tension is generated across the cell-cell adhesions (CCAs), how the dynamic behavior of CMAs may contribute to this, and the exact role tension plays in driving CIL remain unclear.

Here we use *Xenopus* cranial NC cells, an embryonic cell population that exhibits CIL, to elucidate the dynamic behavior of traction forces and CMAs. We show that CMAs, and consequently traction forces, are rapidly reduced near the cell-cell contact upon a collision, generating tension across the cell contact that is required to produce sufficient force to break the cell-cell contact and induce separation during CIL.

## Results

### Traction Forces Are Reduced near the Contact upon a Collision while Tension Builds Up across the Cell-Cell Contact

NC cells exhibit CIL and can be used as a paradigm to study this phenomenon (Carmona-Fontaine et al., 2008, Scarpa et al., 2013). When two NC cells collide they initially collapse their protrusions toward the contact (Figures 1A and 1B green line drop after beige bar). The cells then repolarize and start forming new protrusions away from the contact as they start to pull apart from each other (Figure 1B, red line), causing a thinning of the contact (Figure 1B, blue line), before finally separating and migrating away from each other (Mayor and Carmona-Fontaine, 2010).

In order to elucidate the role of physical forces exerted by cells, we investigated the dynamic behavior of traction forces during CIL by traction force microscopy (TFM), as has been previously described (Scarpa et al., 2015, Theveneau et al., 2013). Single migrating NC cells exerted equal traction at the front and rear of the cell (Figures 1C and 1D); however, an imbalance in traction force is briefly observed when cells start to migrate (Figure 1E). Cells in contact showed significantly reduced traction near the cell-cell contact compared with the free edge (Figures 1F and 1G). This is true for both clusters of cells and colliding doublets of cells. Traction forces of NC cells monitored throughout collision confirmed that traction near the contact is reduced upon collision (Figures 1H and 1I, blue lines). When cells separated after a collision a sudden increase in traction at the rear of the cell was observed (Figures 1J and 1K, blue lines). Taken together, these results show that during CIL there is a suppression of traction force near the cell-cell contact that is alleviated as the cells separate. As cells have to balance the traction generated at the free edge, the reduction of traction at the contact infers a redistribution of intracellular tension toward the CCAs (Figure 1I, red line) that is lost upon separation (Figure 1K, red line) (Maruthamuthu et al., 2011). In order to confirm the existence of tension across the cell-cell contact, laser ablation experiments were carried out on colliding NC cells. Cells were ablated after they started to form protrusions away from the contact (yellow bar in Figure 1B). As NC cells demonstrate a narrowing of the cell-cell contact at the same time as the protrusions are growing away from the contact prior to separation (Scarpa et al., 2015) (Figures 1B and S1B), as determined by extension subtraction analysis (Figure S1G), a shortening in length of the cell-cell contact was used as an indication of cells tending toward separation. NC cells that were not ablated showed a thinning in the length of the cell-cell contact over time and 20% of cells separated within 3 min (Figures 1L, 1Q, and 1R blue; Video S1). When cells were ablated across the p120-catenin labeled CCAs, the cell-cell contact length was rapidly reduced and 70% of cells separated within 3 min (Figures 1M, 1Q, and 1R green; Video S1). This sudden separation supports the hypothesis of tension across the cell-cell contact. TFM demonstrated a reduction in traction near the contact suggesting that CMAs were not transmitting force or contributing to the separation of the cells. To confirm this, cells were ablated in the plane of the CMAs in a region near the cell-cell contact (Figure 1N; Video S1). These cells did not show a significant difference in contact length reduction compared with cells that were not ablated and had a similar separation percentage (Figures 1Q and 1R red). As a control for photodamage, cells were ablated in the cytosol or in the membrane at the free edge. These controls showed no difference in contact length or separation compared with unablated cells (Figures 1O–1R, orange and magenta; Video S2). Furthermore, no difference in survival rate was observed between ablated or unablated cells. To confirm that the conditions used to ablate the CMAs were indeed sufficient to destroy them, CMAs were ablated in protrusions and recoil was observed, indicating a loss of CMAs in this region (Figures S1C and S1D; Video S3). Together, these results indicate that tension is built up across the cell-cell contact during a collision. The lack of traction near the cell-cell contact suggests CMAs may only be present at very low levels in this region and consequently they are unlikely to contribute to the tension across the contact.

Video S1. NC Cells No Ablation, NC Cells Ablated at the CCA and NC Cells Ablated at the CMAs near the Contact, Related to Figure 1 NC cells expressing membrane red fluorescent protein (RFP) (red) and p120-catenin-GFP (green) or GFP-FAK (green). Cells imaged on an Olympus FV1000 microscope using a 60× lens. Frames taken every 30 s. CCA ablated or CMA ablated for 30 s from t = 0 min, area illustrated by cyan box on second repeat. Cells imaged on an Olympus FV1000 microscope using a 60× lens. Frames taken every 30 s

Video S2. Control Ablation in the Cytosol or Free Membrane, Related to Figure 1 NC cells expressing membrane RFP. Cytosol or free membrane ablated for 30 s from t = 0 min, area illustrated by cyan box on second repeat. Cells imaged on an Olympus FV1000 microscope using a 60× lens. Frames taken every 30 s

Video S3. CMA Ablation in the Protrusion of Control Cell, Related to Figure 1 NC cell expressing membrane RFP (red) and GFP-FAK (green). CMAs ablated in the protrusions for 30 s from t = 0, (marked by asterisk), area of ablation illustrated as yellow box on second repeat. Cell imaged on an Olympus FV1000 microscope using a 60× lens. Frames taken every 30 s

### CMAs Disassemble near the Cell-Cell Contact upon a Collision

**Figure 2:**
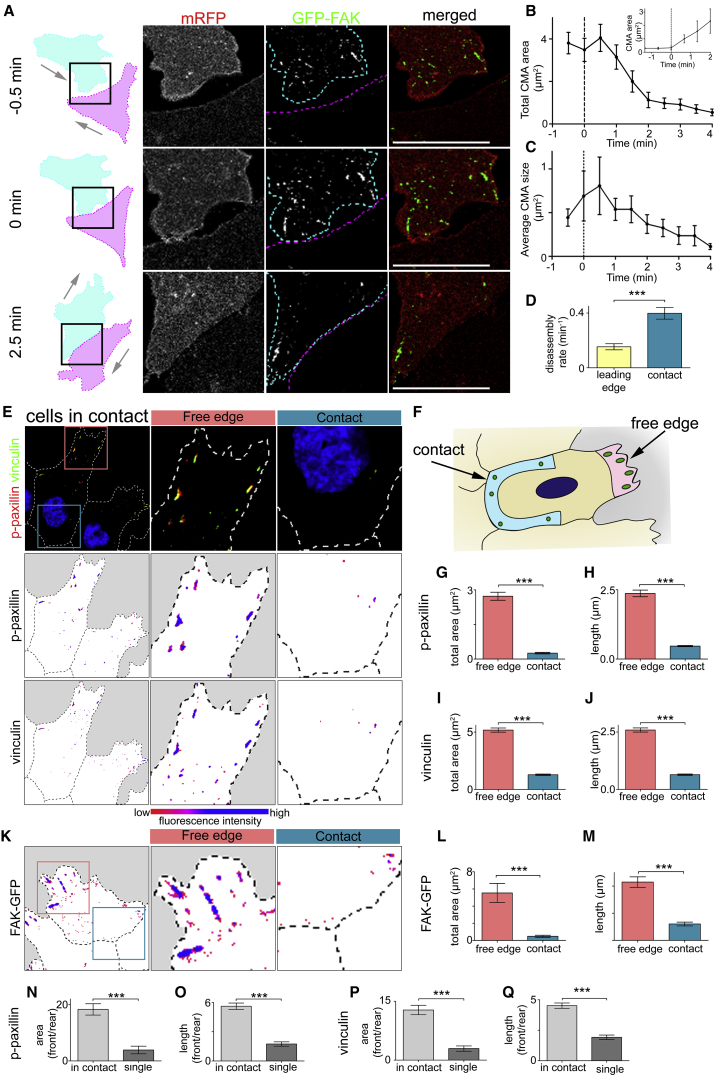
Cell-Matrix Adhesions Disassemble near the Contact upon Collision (A) Outline of colliding cells showed in zoom of contact area between two cells. Cells outlined in cyan and magenta. The cell labeled in cyan is expressing membrane RFP (mRFP) (red) and GFP-FAK (green). (B and C) Total area (B) and average size (C) of CMAs near the contact upon a collision. 0 min = first frame in contact. n = 15 cells. (B insert) Total area of CMAs near the contact as cells separate. 0 min = last frame in contact n = 3 cells. (D) Disassembly rate of CMAs as indicated. Leading edge, n = 24; contact, n = 27. (E and K) Immunocytochemistry on cells in contact against p-paxillin and vinculin (E) or cells expressing FAK-GFP (K) as indicated. Bottom: color indicating fluorescence intensity. (F) Schematic illustrating how regions of interest were defined for analysis. Contact region illustrated in blue and free edge in pink. (G–J, L, and M) Total area and length of CMAs using the markers indicated. n = 80 cells for (G) and (H); n = 76 cells for (I) and (J); n = 16 cells for (L) and (M). (N–Q) CMA polarity determined by ratio of total area (or length) of p-paxillin (N and O) or vinculin (P and Q) labeled CMAs in free edge over contact or leading edge over trailing edge. Scale bars, 20 μm. Line graphs and box graphs show mean, error: ± SEM. ^∗∗∗^p ≤ 0.001. All Mann-Whitney test. See also Figure S2.

Different regions of the cells were identified as described in Figures S1E and S1F and STAR Methods. To visualize the dynamic behavior of CMAs during a collision, NC cells were labeled with GFP tagged FAK (focal adhesion kinase) and imaged while undergoing CIL (Figure 2A; Video S4). FAK is a tyrosine kinase and key signaling component of CMAs that is recruited early on during their maturation (Zaidel-Bar et al., 2004). FAK plays a vital role in coordinating signaling downstream of CMAs. From 30 s after a collision CMAs near the contact began to disappear (Figures 2A–2C) and their disassembly rate was increased (Figure 2D). As soon as colliding cells separate a clear increase in CMAs near the former cell-cell contact is observed (Figure 2B insert). To validate the observed reduction in CMAs near the cell-cell contact, we investigated the distribution of p-paxillin, vinculin, and FAK. Paxillin is phosphorylated on tyrosine residue 118, most likely due to targeting by Src/FAK, during the assembly of CMAs and can be used as a marker of dynamic CMAs (Zaidel-Bar et al., 2007). In addition, vinculin is recruited to CMAs where it is involved in crosslinking actin to adapter proteins and responds to tensions across the adhesion complex (Grashoff et al., 2010). NC cells showed a significant reduction of CMAs at the contact (defined as the contact area excluding regions near the free edge; blue region in Figure 2F) compared with those at the free edge (identified as the protrusion region free of yolk platelets, Figure S1E; pink region in Figure 2F), as measured by a reduction in the total area and length of p-paxillin stained CMAs, vinculin stained CMAs, and GFP-FAK labeled CMAs (Figures 2E–2M). While single cells also showed a significant reduction in CMAs at the rear of the cell compared with the front of the cell (Figures S2A–S2F), the CMA polarity (the ratio of CMAs at the front [free edge or leading edge] and at the rear [contact or trailing edge]) was significantly greater for cells in contact (Figures 2N–2Q). This shows that the cytoskeletal polarity exhibited by an isolated migratory cell alone is insufficient to account for the CMA polarity of cells in contact, suggesting a role for the cell-cell contact itself in enhancing CMA disassembly at the contact.

Video S4. CMAs at Contact during CIL, Related to Figure 2 Zoom of a contact of NC cells undergoing CIL. Cells expressing membrane RFP (red) and GFP-FAK (green) and shown in phase contrast (final panel). Cells are in contact from 0 min. Prior to collision, CMAs appeared in the leading edge and continued to grow as the cell migrated. From 30 s after collision, CMAs near the contact (indicated by arrowhead) began to disappear. CMAs away from the site of contact (indicated by asterisk), such as those at the periphery of the cell, persisted during a collision. Cells were filmed on a SP8vis confocal microscope with 63× objective lens. Brightness/contrast optimized for top cell. Frames taken every 30 s

### N-cadherin Regulates CMA Disassembly at the Contact

**Figure 3:**
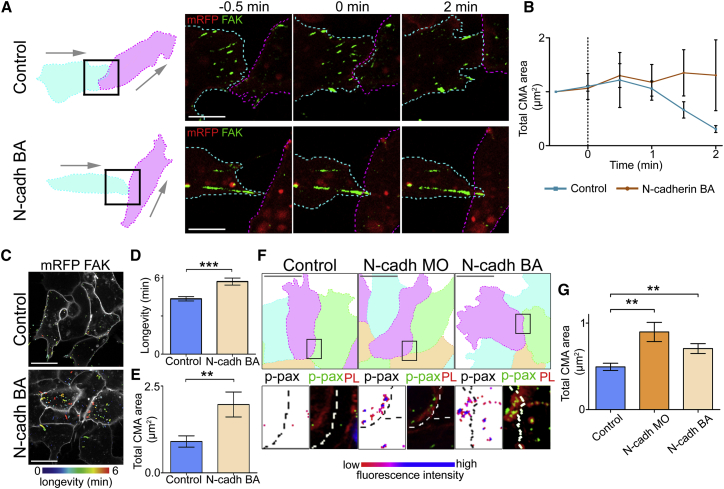
N-cadherin Junction Formation Leads to Cell-Matrix Adhesion Disassembly near the Contact (A) Outline and direction of migration of colliding cell pair, either in the absence or presence of N-cadherin (N-cadh) BA. Cells expressing membrane RFP (red) and GFP-FAK (green). (B) Total area of GFP-FAK labeled CMAs at the contact during a collision. 0 min: first frame of contact, n = 3 collisions for both conditions. (C) Control and N-cadherin BA treated cells expressing membrane RFP (gray) and GFP-FAK color coded for longevity. (D) Longevity of CMAs near contact of cells expressing GFP-FAK. n = 30 for each condition. (E) Total area of FAK labeled CMAs near the contact. n = 28 cells for both conditions. (F and G) (F) Cells in clusters; zoom of region of contact shown by immunocytochemistry against p-paxillin in control cells (n = 171) cells expressing N-cadherin morpholino (n = 89) or treated with N-cadherin blocking antibody (n = 68). p-paxillin (green), phalloidin (red), Hoescht (blue). P-paxillin alone in left zoom colored according to fluorescence intensity (G). Total area of p-paxillin labeled CMAs at the contact. n = 68 cells. Scale bars, 20 μm. Line graphs show median; errors, interquartile range. Bar graphs show mean; errors ± SEM. ^∗∗∗^p ≤ 0.001, ^∗∗^p ≤ 0.01. Mann-Whitney test. See also Figure S3.

Migratory NC cells, solely used in this paper, predominantly express N-cadherin. N-cadherin is known to be essential for CIL between NC cells (Bahm et al., 2017, Theveneau et al., 2010). The inhibition of N-cadherin prevents NC cells from undergoing CIL; instead they continue to walk past each other upon a collision (Theveneau et al., 2010). Furthermore, protrusions are not inhibited at the site of contact as occurs in control collisions (Theveneau et al., 2010, Theveneau et al., 2013) preventing *ex vivo* explants from dispersing as they would in control explants (Theveneau et al., 2010). As the loss of CMAs near the cell-cell contact occurs after N-cadherin recruitment to the contact (Figures S2G and S2H), N-cadherin may play a role in stimulating the disassembly of CMAs. To investigate this, N-cadherin adhesions were inhibited using an N-cadherin blocking antibody (BA) (Hatta and Takeichi, 1986, Theveneau et al., 2013) in GFP-FAK expressing NC cells undergoing a collision. Control NC cells showed a reduction in CMAs upon a collision (Figures 3A and 3B blue line), whereas cells treated with the N-cadherin BA did not (Figures 3A and 3B orange line). Furthermore, cells treated with the N-cadherin BA showed an increase in the longevity of CMAs (Figures 3C and 3D) suggesting the formation of N-cadherin-based adhesions is required to promote the disassembly of CMAs near the contact. Analysis of cells in a cluster demonstrated a significant increase in the total area of CMAs near the contact in cells treated with the N-cadherin BA compared with controls (Figure 3E). Immunostaining against endogenous CMAs validated this observation and this was further supported by a significant increase of CMAs also being observed when N-cadherin expression was inhibited with the use of an N-cadherin morpholino (Figures 3F and 3G). Together these results suggest that N-cadherin is involved in driving the localized disassembly of CMAs near the cell-cell contact during CIL.

### Loss of N-cadherin Function Decreases Src Activity

**Figure 4:**
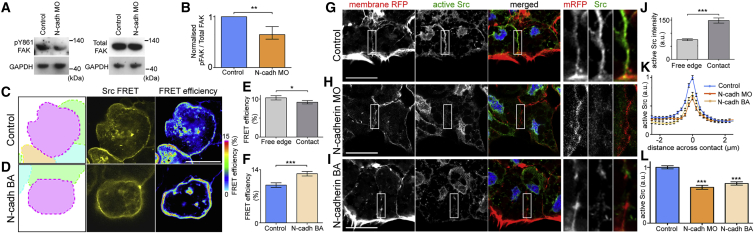
N-cadherin Binding Leads to Src Activity at the Contact (A) Western blot against total FAK and pY861 FAK in control or N-cadherin MO cells. (B) Quantification of (A); n = 3 repeats. (C and D) Cell clusters for Src-FRET as indicated. (E and F) Src-FRET efficiency as indicated. n = 23 cells for each condition. (G–I) Immunocytochemistry against active Src (green) in cells injected with membrane RFP (red) and stained with Hoescht (blue) as indicated. Box shows region of zoom over contact. (J–L) Intensity of active Src at contact and free edge as indicated. Control, n = 125; N-cadh MO, n = 60; N-cadh BA, n = 64. Scale bar, 20 μm. Line graph shows means, errors ± SEM; bar graphs show means; error bars are ±SEM. ^∗∗∗^p ≤ 0.001, ^∗∗^p ≤ 0.01, ^∗^p ≤ 0.05. (B, F, and L) t test, (E and J) paired t test.

The non-receptor tyrosine kinase Src is known to regulate CMA disassembly (Webb et al., 2004). Src can be recruited to adherens junctions (Tsukita et al., 1991) and activated downstream of cadherins (Leonard et al., 2013, McLachlan et al., 2007, Shao et al., 2016, Truffi et al., 2014). We investigated Src as a possible candidate involved in the observed crosstalk between N-cadherin and CMAs during CIL. First, a functional readout of Src activity was carried out by measuring phosphorylation of FAK on a Src-specific residue Tyr861 by western blot analysis on lysates of whole *Xenopus laevis* embryos (Calalb et al., 1996). When N-cadherin expression was reduced by morpholino oligomer (MO) injection in embryos, a significant reduction in FAK pY861 was observed, representing a decrease in Src activity (Figures 4A and 4B). Second, a Src-fluorescence resonance energy transfer (FRET) probe was used to visualize Src activity, in which FRET efficiency is inversely related to Src activity (Wang et al., 2005). In control cells FRET efficiency was significantly reduced at the cell-cell contact compared with the free edge, suggesting an increase in Src activity at the contact compared with the free edge (Figures 4C and 4E). The inhibition of N-cadherin, using the N-cadherin BA, led to a significant increase in FRET efficiency compared with controls (Figures 4C, 4D, and 4F), confirming that the loss of N-cadherin-based adhesions results in reduced Src activity. Third, immunofluorescence against active Src (Kawakatsu et al., 1996) revealed a clear recruitment of active Src to the contact between two cells; active Src levels at the contact of control cells were significantly higher than active Src levels at the free edge (Figures 4G and 4J). However, when N-cadherin was perturbed either with the use of an antisense MO or the BA, a significant reduction in active Src levels at the cell contact was observed (Figures 4H–4L). Together, these results demonstrate that N-cadherin is involved in the activation of Src at the contact.

### Inhibition of Src/FAK Signaling Increases CMAs at the Contact

**Figure 5:**
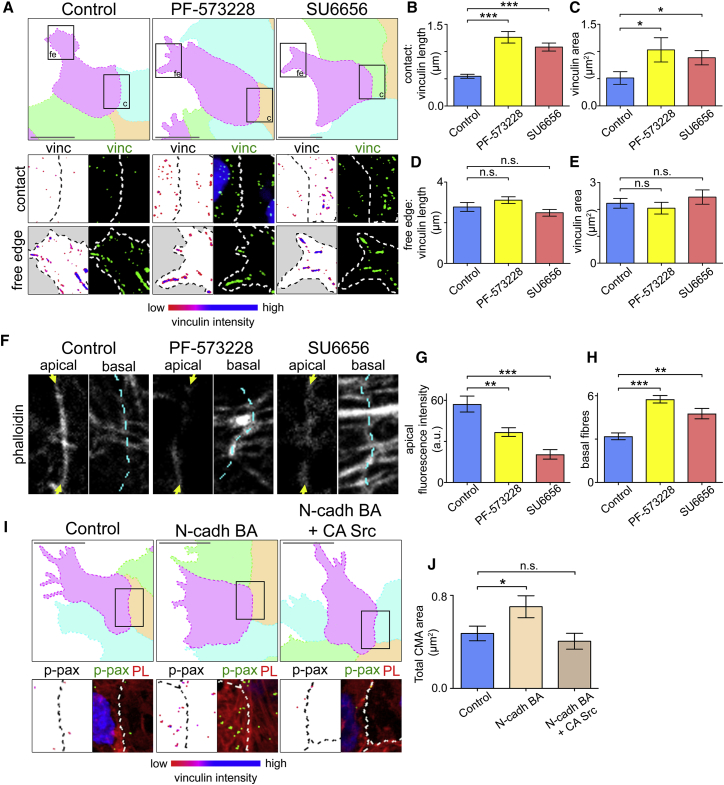
Src/FAK Inhibition Increases Cell-Matrix Adhesions near the Contact (A and I) Immunocytochemistry against vinculin as indicated. Zoom of region of contact and free edge region shown in black boxes. Vinculin shown as green in merged image with Hoescht (blue) and cell outlines. Vinculin alone in zoom colored according to fluorescence intensity. (B–E) Length and total area of vinculin labeled CMAs. Control, n = 33; PF-573228, n = 24; SU6656, n = 36 cells. (F–H) Phalloidin staining and quantification as indicated. Arrows indicate phalloidin staining; dotted line indicates contact between adjacent cells. (I) Immunocytochemistry against p-paxillin as indicated. Zoom of region of contact shown in black box. p-paxillin shown as green in merged image with phalloidin (red), Hoescht (blue), and cell outlines. P-paxillin alone in zoom colored according to fluorescence intensity. (J) Total CMA area per cell near the contact. Control, n = 52; N-cadh BA, n = 50; N-cadh BA + Src Y527F, n = 49 cells. Scale bars, 20 μm. Bar graphs show mean, error bars are ±SEM. ^∗∗∗^p ≤ 0.001, ^∗∗^p ≤ 0.01, ^∗^p ≤ 0.05. All Kruskal-Wallis tests. See also Figure S4.

Src is a key regulator of CMA dynamics. One known mechanism by which Src regulates CMA dynamics is through the tyrosine kinase FAK (Xing et al., 1994). Src activates FAK at the CMAs and together they can promote the disassembly of CMAs (Myers and Gomez, 2011, Webb et al., 2004, Westhoff et al., 2004, Woo et al., 2009). To confirm the effect of Src and FAK inhibition on CMAs, the small molecule inhibitors SU6656 (Blake et al., 2000) and PF-573228 (Slack-Davis et al., 2007) were used to inhibit Src and FAK kinase activity respectively. Inhibiting either Src or FAK kinase activity resulted in an increase in the length and area of the vinculin labeled CMAs near the contact compared with control cells (Figures 5A–5C), without significantly affecting the CMAs in the free edge (Figures 5A, 5D, and 5E), and increased the stabilization of CMAs in treated cells (Figures S3A and S3B). To identify whether the stabilization of CMAs resulted in changes to actin behavior, apical and basal actin pools were visualized using phalloidin. When CMAs were stabilized with either Src inhibitor or FAK inhibitor treatments, the number of basal stress fibers near the cell-cell contact increased compared with controls, whereas the levels of apical actin at the cell-cell contact were reduced (Figures 5F–5H).

To confirm that the effect of Src is downstream of N-cadherin, N-cadherin was inhibited by applying the BA to cells expressing constitutively active Src (Sandilands et al., 2004, Timpson et al., 2001). The expression of constitutively active Src was able to rescue the increase in CMAs observed near the cell-cell contact in cells where N-cadherin was perturbed and reduce the CMAs back to control levels (Figures 5I and 5J). Furthermore, when cells were plated on a mixed substrate of fibronectin and N-cadherin (N-cadherin Fc chimera protein), a reduction in CMAs was observed compared with cells plated on fibronectin only (Figures S3C, S3D, and S3I). This reduction could be rescued by incubating the cells with the FAK or Src inhibitor (Figures S3E, S3F, and S3I). Cells incubated in the N-cadherin BA prior to plating, where the formation of N-cadherin-based adhesions were prevented, did not show a significant reduction in CMAs compared with controls, confirming the specificity of N-cadherin in the substrate in inducing the observed reduction in CMAs (Figures S3G and S3I). To verify the specificity of p-paxillin in labeling the integrin-based adhesions and exclude the possibility of p-paxillin labeling N-cadherin-based adhesions, cells were plated on an N-cadherin substrate alone. These cells did not form p-paxillin labeled CMAs (Figures S3H and S3I).

Together these results demonstrate that inhibiting the kinase activity of either Src or FAK leads to an increase in CMAs near the contact and therefore suggest that Src and FAK facilitate the disassembly of CMAs during CIL. In addition, the results confirm that FAK-Src signaling works downstream of N-cadherin in driving the observed reduction in CMAs near the cell-cell contact.

### Src/FAK Inhibition Reduces NC Migration *In Vivo* and Dispersion

**Figure 6:**
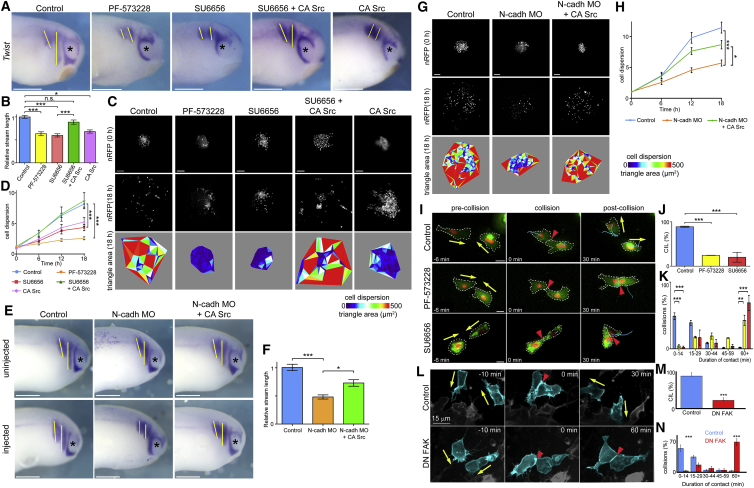
FAK-Src Signaling Required for NC Dispersion, Migration, and CIL (A) Migrating NC labeled with *twist* in embryos at stage 24, after the indicated treatments. Yellow lines, distance of NC migration; asterisks, eye. Scale bar, 500 μm. (B) Length of second NC stream relative to mean control of each experiment. Control n = 101, PF-573228 n = 47, SU6656 n = 46, SU6656 + CA Src n = 35, CA Src n = 7 embryos. (C) Initial frame (0 hr) and last frame (18 hr) from movies of explanted NC cell clusters expressing nuclear RFP, after the indicated treatments. Dispersion analysis based on Delaunay triangles color coded for area size. Scale bar: 100 μm. (D) Normalized mean triangle areas over time. Control n = 52, PF-573228 n = 28, SU6656 n = 32, SU6656 + Src Y527F n = 32, CA Src n = 10 explants. (E) Migrating NC labeled with *twist* in embryos at stage 24, after the indicated treatments. Yellow lines, distance of NC migration; asterisks, eye. Scale bar, 500 μm. (F) Length of second NC stream migration of injected side relative to uninjected side. n = 33 embryos for control, n = 30 embryos for N-cadherin MO injected embryos, and n = 28 for embryos co-injected with N-cadherin MO and constitutively active Src. (G) Initial frame (0 hr) and last frame (18 hr) from movies of explanted NC cell clusters expressing nuclear RFP, after the indicated treatments. Dispersion analysis based on Delaunay triangles color coded for area size. Scale bar, 100 μm. (H) Normalized mean triangle areas over time. n = 24 explants for all conditions. (I) Frames from movies of colliding pairs of NC cells expressing membrane GFP (green) and nuclear RFP (red), after the indicated treatments. Yellow arrows, direction of migration; red arrowhead, cell collision. Scale bar, 10 μm. (J) CIL within 30 min of colliding, n = 3 repeated experiments for PF-573228 and SU6656. Number of collisions analyzed: control = 250, PF-573228 = 127, SU6656 = 74. (K) Duration of contact; three repeated experiments for PF-573228 and SU6656. Number of collisions analyzed: control = 250, PF-573228 = 127, SU6656 = 74. (L) *In vivo* time-lapse imaging of Sox10-GFP zebrafish embryos showing CIL between NC cells. Yellow arrows, direction of migration; red arrowhead, cell collision; n = 35 embryos. (M) CIL *in vivo* within 30 min of colliding, n = 5 repeated experiments for control and DN F FAK. Number of collisions analyzed: control = 150, DN FAK = 117. (N) Duration of contact. Five repeated experiments for control and DN FAK. Number of collisions analyzed: control = 150, DN FAK = 117. Line graphs and bar graphs show mean ± SEM. ^∗∗∗^p ≤ 0.001, ^∗∗^p ≤ 0.01, ^∗^p ≤ 0.05. All ANOVA test. See also Figures S4 and S5.

The results presented so far have focused on the behavior of *ex vivo* NC explants. In order to establish whether inhibition of either Src or FAK affects NC migration *in vivo*, embryos were incubated in the respective inhibitors and NC migration was analyzed. Embryos incubated with either the Src or FAK inhibitor, or where constitutively active Src was expressed, showed reduced NC migration compared with control (Figures 6A and 6B). The reduction in migration caused by the Src inhibitor could be rescued by the expression of constitutively active Src (Figures 6A and 6B green bar), indicating the specificity of the Src inhibitor. To corroborate this result, both *Xenopus* and zebrafish embryos were made to express FRNK, a truncated form of FAK that works as a dominant negative. Analysis of NC migration was performed in *Xenopus* by performing *in situ* hybridization against the NC marker *twist*, whereas time-lapse imaging was performed in zebrafish, using the Sox10-GFP transgenic line that expresses GFP in the NC cells (Moore et al., 2013). A significant reduction in NC migration was observed in both *Xenopus* and zebrafish embryos expressing the dominant-negative form of FAK (Figure S4).

NC explants disperse when cultured *ex vivo* (Alfandari et al., 2003), CIL being the driving force behind this dispersion (Carmona-Fontaine et al., 2011, Davis et al., 2012, Smeets et al., 2016). When NC explants were incubated with either the Src or FAK inhibitors, or were expressing constitutively active Src, the dispersion of the explants was significantly reduced compared with the control clusters (Figures 6C and 6D). The reduction in dispersion caused by Src inhibition could once again be rescued by expression of constitutively active Src (Figures 6C and 6D green line). These results demonstrate that inhibition of Src and FAK indeed perturb *in vivo* NC migration during development and suggest that they do so by affecting CIL.

N-cadherin is required for CIL and consequently for the collective directional migration of the NC (Theveneau et al., 2010). *Xenopus* embryos co-injected with N-cadherin MO and constitutively active Src demonstrated a partial rescue; NC migration was significantly improved *in vivo* compared with embryos injected with N-cadherin MO alone (Figures 6E and 6F). Furthermore, NC explants injected with both the N-cadherin MO and constitutively active Src demonstrated a rescue in their dispersion *in vitro* (Figures 6G and 6H). These results highlight the importance of Src activation downstream of N-cadherin in facilitating NC migration.

### Src and FAK Inhibition Perturbs CIL

To verify the role of Src and FAK in CIL, cell collisions were assessed. When control NC cells collided they underwent CIL in almost 90% of cases (Figures 6I and 6J, blue bar; Video S5). In contrast, NC cells incubated in either the Src or FAK inhibitor showed a dramatic decrease in CIL response (Figures 6I and 6J, yellow and red bars; Video S5). Analysis of contact durations revealed that while the majority of control cells separated within 15 min after a collision, most of the Src or FAK inhibited cells had failed to separate 60 min after colliding (Figure 6K). To corroborate this result *in vivo*, CIL was visualized in NC cells by time-lapse imaging using the Sox10-GFP transgenic zebrafish embryo. NC cells expressing a dominant-negative form of FAK fail to separate after a collision in a manner that closely mimics the *ex vivo* result (Figures 6L–6N).

Video S5. CIL Collision Assay of Control and Src or FAK Inhibited Cells, Related to Figure 6 Live imaging of NC cells undergoing CIL. Conditions: control, treatment with PF-573228 or SU6656. Cells express nuclear RFP (red) and membrane GFP (green) and also imaged with brightfield. All cells collide at 0 min. Cell tracks are overlaid in blue and red. Cells imaged on a DMRXA2 Leica Microscope with a 20× objective lens. Frames taken every 3 min

**Figure 7:**
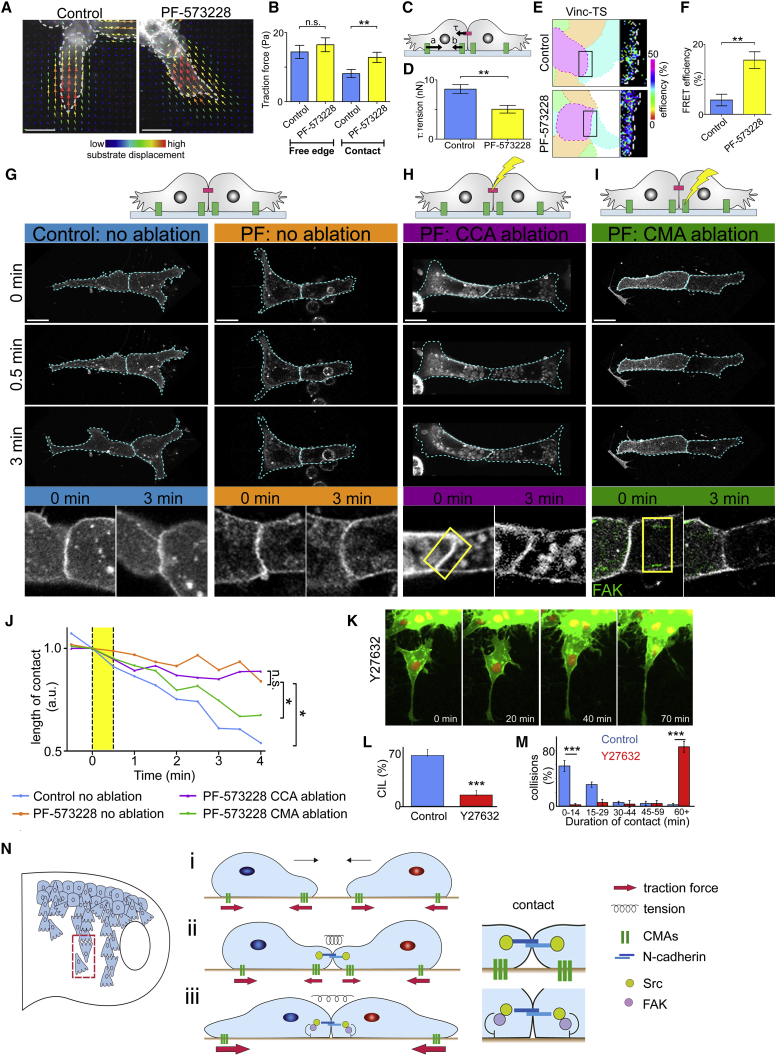
Disassembly of Cell-Matrix Adhesionss at the Contact Is Required for Separation. (A) TFM of cells expressing membrane RFP (gray) outlined (gray) and substrate displacement vectors color coded to their magnitude. Control and PF-573228 (FAK inhibitor) treated cells. (B) Traction force magnitude at the free edge and at the contact. n = 24 cells for both conditions. (C) Schematic of tension across cell-cell contact inferred from the balance of forces: traction at free edge (a), traction near the contact (b), tension across the contact (τ). (D) Tension across the contact. Control, n = 39; PF-573228, n = 18 cells. (E) FRET efficiency at cell-cell contact from vinculin-tensor sensor in control and PF-573228 treated cells. (F) FRET efficiency at the cell-cell contact. (G–I) Schematic of cells under the indicated ablations. Below: frames from movies after collisions. Cells expressing membrane RFP (gray) and GFP-FAK (green in zoom). Zoom of cell-cell contact. Ablation area marked with a yellow box on zoom. To maintain the same orientation and scale, images in G–I were rotated, zoomed, and the background was filled with black. (J) Length of the cell-cell contact relative to the start of laser ablation (0 min). Yellow area shows time of laser ablation. n = 15 doublets for control no ablation (blue), PF-573228 no ablation (orange), and PF-573228 CCA ablation (purple). PF-573228 CMA ablation n = 18 doublets (green). (K) Cells expressing membrane GFP and nuclear RFP treated with the ROCK inhibitor Y-27632. (L) Percentage of cells that undergo CIL after colliding. (M) Duration of contact; 87 cell collisions analyzed in four independent experiments. (N) Model summarizing main results. Red dotted rectangle is shown as zoom in i, ii, and iii. Scale bars, 20 μm. Bar graphs show means, errors ± SEM. Line graph shows medians. ^∗∗∗^p ≤ 0.001, ^∗∗^p ≤ 0.01, ^∗^p ≤ 0.05. (B and D) Mann-Whitney test, (F and L) t test, (J) ANOVA test.

To address why cells failed to separate, a number of possibilities were investigated. First, single NC cells in which either Src or FAK was inhibited were still migratory (Video S6) and therefore lack of separation is unlikely to result from an inability of cells to migrate. Second, an increase in CCA could perturb separation after a collision. Using two cell adhesion assays (Kashef and Franz, 2015), no significant difference in CCA strength was observed between control cells and those treated with either the Src or FAK inhibitor when cells were plated on an NC monolayer (Figures S5A and S5B) or an N-cadherin substrate (Figures S5C and S5D). However, cells injected with the N-cadherin MO showed significantly more detachment than control cells (Figures S5A–S5D), and overexpressing N-cadherin showed a significant increase in attachment to N-cadherin substrate (Figure S5D), demonstrating the sensitivity of these assays. Migratory NC cells predominantly express N-cadherin; however, E-cadherin is also still present at low levels at this stage. The endogenous levels of both N-cadherin and E-cadherin across cell-cell contacts were found to be unaffected by the inhibition of FAK or Src (Figures S5E–S5H). Together, these results support the idea that the loss of separation is not caused by an increase in CCA. Third, protrusion formation away from the contact is required for separation during CIL in NC cells (Scarpa et al., 2015); therefore, free edge protrusions were analyzed in control and treated cells. Protrusions formed at the free edge in cells treated with either the Src or FAK inhibitor in a manner that mirrored the dynamic behavior of the protrusions in control cells (Figures S5I and S5J). In addition, traction forces generated at the free edge of cells treated with either the Src or FAK inhibitor were the same as controls (Figures 7A and 7B). These results suggest that free edge protrusions are not altered by inhibition of Src and FAK and are therefore unlikely to be contributing to the lack of separation that occurs in cells treated with the Src or FAK inhibitor.

Video S6. Control and Treated Cells Migrating, Related to Figure 6 Brightfield images of single NC cells migrating. Conditions either control, or treated with PF-573228 (FAK inhibitor) or SU6656 (Src inhibitor). Cell tracks are overlaid in blue and red. Cells imaged on a DMRXA2 Leica Microscope with a 20× objective lens. Frames taken every 3 min

Finally, traction force generated near the cell-cell contact was found to be increased in cells where FAK was inhibited (Figures 7A and 7B contact), and the corresponding tension across the cell-cell contact, inferred from the traction forces (Maruthamuthu et al., 2011), was reduced (Figures 7C and 7D). To confirm this result, tension across the contact was measured using a FRET-based vinculin-tensor sensor (Grashoff et al., 2010, Kuriyama et al., 2014). FRET efficiency across the contact was increased in FAK inhibited cells, confirming a reduction in tension when FAK is inhibited (Figures 7E and 7F). Decreased tension across cell-cell contact could explain the lack of separation seen in FAK inhibited cells.

### Disassembly of CMAs at the Contact Is Required for Separation during CIL

We hypothesize that stabilization of CMAs near the cell-cell contact in cells where Src or FAK is inhibited is preventing the buildup of tension across the cell-cell contact required for cell separation and thus inhibiting CIL. According to this hypothesis the physical removal of CMAs near the contact in FAK inhibited cells should rescue the FAK inhibition phenotype and lead to cell separation. To test this hypothesis, CMAs were physically disrupted at the contact of colliding cells by laser ablation after the cells repolarized while monitoring the length of cell-cell contacts. In the absence of ablation, contact length of FAK inhibited cells remained relatively constant (Figures 7G and 7J orange line; Video S7), while the contact length of control cells steadily reduced over time (Figures 7G and 7J blue line; Video S7). To verify that separation was not prevented by a stronger CCA in cells where FAK was inhibited, the cell-cell contact was ablated. Contact length in these ablated cells showed no significant difference compared with the non-ablated cells (Figures 7H and 7J purple line; Video S8), demonstrating that CCA is not the cause of cells remaining together after FAK inhibition. However, when CMAs near the contact of FAK inhibited cells were ablated, the length of the cell-cell contact decreased to levels similar to those seen in control cells (Figures 7I and 7J green line; Video S9). Both CMAs and CCAs demonstrated quick recovery after ablation in the absence of separation. Overall, these results indicate that the presence of increased CMAs at the contact in FAK inhibited cells prevents cell separation. These results suggest that during CIL the disassembly of CMAs near the contact is required in order to redistribute tension generated across the cell to the cell-cell contact to initiate cell separation. To test whether tension across the contact is required for cell separation, we inhibited contractility by treating the cells with the ROCK inhibitor Y-27632. The majority of cells where contractility was inhibited did not separate within the first hour after a collision, whereas the majority of control cells separated within the first 15 min after a collision (Figures 7K–7M). This confirms the importance of tension across the contact to drive separation after a collision.

Video S7. Control and FAK Inhibited Cells with No Ablation, Related to Figure 7 NC cells expressing membrane RFP. Either control (left) or FAK inhibited (PF-573228, right). Cells imaged on an Olympus FV1000 microscope using a 60× lens. Frames taken every 30 s

Video S8. Ablation of CCAs of FAK Inhibited Cells in Contact, Related to Figure 7 NC cells expressing membrane RFP (gray). Cells were treated with PF-573228 to inhibit FAK. CCA ablated for 30 s from t = 0, illustrated as yellow box on second repeat. Cells imaged on an Olympus FV1000 microscope using a 60× lens. Frames taken every 30 s

Video S9. Ablation of CMAs of FAK Inhibited Cells in Contact, Related to Figure 7 NC cells expressing membrane RFP (red) and GFP-FAK (green). Cells treated with PF-573228 to inhibit FAK. CMAs ablated near the contact for 30 s from t = 0, illustrated as yellow box on second repeat. Cell imaged on an Olympus FV1000 microscope using a 60× lens. Frames taken every 30 s

## Discussion

CIL is a dynamic multi-step process that drives the inhibition of protrusions at the site of contact between cells and promotes the formation of protrusions away from the contact, eventually leading to the separation of the cells. There are many different molecular mechanisms and components that contribute to this migratory phenomenon (Roycroft and Mayor, 2016, Stramer and Mayor, 2017).

The results presented here have helped establish the sequence of events during CIL and have identified a redistribution of adhesive forces within colliding cells. Before collision, freely migrating single cells exhibit CMAs both at their leading and trailing edges with equal traction forces at the front and rear of the cell (Figure 7Ni). During collisions, cells establish a functional N-cadherin-based adhesion, which in turn activates Src (Figure 7Nii). Src, through FAK, enhances the disassembly of CMAs near the contact, reducing traction in this region and consequently redirecting the traction generated at the free edge of the cell to the cell-cell contact (Figure 7Niii). This intracellular redistribution of forces is essential for initiating cell separation during CIL.

Although initially discussed in the 1970s (Abercrombie, 1970, Abercrombie and Dunn, 1975, Harris, 1973), the importance of CMAs during CIL has been largely overlooked. With the help of advanced imaging techniques, we demonstrate here a rapid disassembly of CMAs near the cell-cell contact upon a collision that is driven by the formation of N-cadherin-based CCAs. Crosstalk between CCAs and CMAs has previously been demonstrated in various systems (Collins and Nelson, 2015, McMillen and Holley, 2015, Mertz et al., 2013, Mui et al., 2016, Weber et al., 2011), with the presence of one often leading to the localized downregulation of the other (Burute and Thery, 2012, Wilson, 2011). For example, in astrocytes N-cadherin is associated with the suppression of CMA formation (Camand et al., 2012), and the presence of N-cadherin in the paraxial mesoderm mesenchyme in zebrafish has been shown to locally inhibit CMAs (Jülich et al., 2015). Here we demonstrate for the first time that a similar crosstalk can lead to CIL and cell separation in the NC.

We show that N-cadherin downregulates CMAs through the tyrosine kinase Src. The recruitment and activation of Src downstream of cadherins has previously been demonstrated in other systems (Leonard et al., 2013, McLachlan et al., 2007, Shao et al., 2016, Tsukita et al., 1991, Veracini et al., 2015), and a similar mechanism seems to be occurring in the NC. One known role of Src is in the activation of the CMA protein FAK (Calalb et al., 1995), which consequently exposes a binding site for Src itself. This allows the formation of a FAK-Src complex (Xing et al., 1994) and leads to further phosphorylation of FAK in the kinase loop (Owen et al., 1999). Here we show that Src/FAK signaling is required for the normal disassembly of CMAs as inhibition of Src/FAK activity leads to reduced turnover and increased stability of the CMAs. The importance of Src and FAK in driving CMA disassembly has been known for some time (Fincham and Frame, 1998, Myers and Gomez, 2011, Nakamura et al., 1993, Webb et al., 2004, Westhoff et al., 2004, Woo et al., 2009), and previous findings have shown that cells lacking FAK or Src activity have enlarged CMAs due to a reduction in disassembly (Chen et al., 2002, Ilić et al., 1995). How Src/FAK activity leads to CMA disassembly is not fully understood and many different mechanisms have been implicated, including Rho contractility (Chen et al., 2002), calpain proteolysis (Carragher et al., 2003), and microtubule dynamics (Ezratty et al., 2005). However, which of these, or other mechanisms (Collins and Nelson, 2015), are playing a role in CMA disassembly near the point of contact during CIL remains to be uncovered.

The work presented here highlights the role of tension across the cell-cell contact in inducing cell separation. Although the roles of intercellular forces during CIL were discussed early on during their discovery (Abercrombie, 1970, Abercrombie and Ambrose, 1958), it is only in recent years that tension has been shown to build up across the cell-cell contact as a consequence of repolarization and the coupling of the cytoskeletons across the cell-cell junction during normal CIL (Davis et al., 2015, Scarpa et al., 2015). When the buildup of tension is perturbed, for example by preventing repolarization or inhibiting stress fibers across the contact, the cells no longer separate during CIL, indicating a requirement for tension across the cell-cell contact for driving separation (Davis et al., 2015, Scarpa et al., 2015). Here we show that repolarization and consequently traction away from the contact is insufficient to promote cell separation and that the generated tension has to act through the cell-cell contacts in order to initiate the separation process. Colliding cells reduce their CMAs and consequently traction near the site of contact. Since traction at the trailing (free) edge is maintained, tension generated by the cells is transferred to the cell-cell contacts. Here we show that this redistribution of forces within the cell is a fundamental part of CIL. It leads to the generation of asymmetric traction forces within the individual cells that promotes separation by increasing tension across the cell-cell contact.

In conclusion, our results demonstrate that separation during CIL requires a reduction of CMAs near the contact upon collision in order to allow the transfer of tension to the CCA for consequent separation. The reduction of CMAs is dependent upon N-cadherin and its ability to activate Src and subsequently FAK, leading to the disassembly of CMAs. NC cells lacking N-cadherin, or Src or FAK kinase activity, cannot separate during CIL due to the stabilization of CMAs near the contact and the consequent increase in traction, which in turn prevents buildup of tension across the cell-cell contact. CIL occurs within a wide range of cell types and contributes to several morphogenetic processes, making it one of the fundamental cellular mechanisms during development and disease. The proposed mechanism herein sheds a new light on cell-cell interactions governed by CIL, therefore opening the door to novel implications in the role CIL plays in development and cancer invasion.

## STAR★Methods

### Key Resources Table

**Table:** 

REAGENT or RESOURCE	SOURCE	IDENTIFIER
**Antibodies**		

anti-Phospho-paxillin pY118	ThermoFisher	44-722G
anti-vinculin	Sigma-Aldrich	V9131
anti-N-cadherin	DSHB	MNCD2
Anti-active Src clone 28	ThermoFisher	AHO0051
anti-E-cadherin	DSHB	5D3
N-cadherin blocking antibody – CDH2	ThermoFisher	13-2100

**Chemicals, Peptides, and Recombinant Proteins**		

Src inhibitor – SU6656	Sigma-Aldrich	S9692
FAK inhibitor – PF-573228	Sigma-Aldrich	PZ0117
N-cadherin Fc chimera protein	R&D systems	6626-NC
Fibronectin	Sigma-Aldrich	F1141
Phalloidin Rhodamine - TRITC	Sigma-Aldrich	P2141

**Experimental Models: Organisms/Strains**		

*Xenopus laevis*	European Xenopus Research Centre	N/A

**Oligonucleotides**		

N-cadherin morpholino antisense oligos ‘5′-GAAGGGCTCTTTCCGGCACATGGTG-3’	Gene Tools as described in (Theveneau et al., 2010)	N/A

**Recombinant DNA**		

Plasmid: FAK-GFP	Gomez Lab at University of Wisconsin as described in (Myers and Gomez, 2011)	N/A
Plasmid: constitutively active Src Y527F	Yap Lab at University of Queensland, Australia	N/A
Plasmid: P120-catenin GFP	(Scarpa et al., 2015)	N/A
Plasmid: N-cadherin - RFP	(Scarpa et al., 2015)	N/A
Plasmid: Src FRET biosensor	Wang Lab at UC San Diego as described in (Wang et al., 2005)	N/A

**Software and Algorithms**		

Traction force microscopy analysis plugin	This paper	N/A
Focal Adhesion Analysis Server	Developed by the Gomez Lab at Universtiy of North Carolina. (Berginski and Gomez, 2013)	faas.bme.unc.edu
Image J	NIH	N/A
PRISM	GraphPad	N/A

### Contact for Reagent and Resource Sharing

Further information and requests for resources and reagents should be directed to and will be fulfilled by the Lead Contact, Roberto Mayor (r.mayor@ucl.ac.uk).

### Experimental Model and Subject Details

Embryos were obtained from adult animals from the species *Xenopus laevis* via *in vitro* fertilization. Embryos were maintained in NAM in an incubator between 14°C and 20°C as previously described (Bahm et al., 2017). Sex/gender was not assessed due to its irrelevance in this study at these early embryonic stages. The animal procedures were mild and they were performed under a Home Office licence. Adult animals were kept at 18 C in Techniplast Aquatic system with recirculated water and biological filter, with dark-light cycles of 12 hours. Oocytes were obtained by hormone induced ovulation and collected in 10X NAM.

### Method Details

#### Embryos, Microinjection and Dissection

*Xenopus laevis* embryos were staged according to Nieuwkoop and Faber method, 1967; briefly, embryos were examined under a stereomicroscope and their morphological features were compared with a normal *Xenopus laevis* developmental table. They were injected with either mRNA or DNA at the 4 or 8 cell stage. Embryos were injected in Ficoll solution (Sigma), a highly branched hydrophilic polysaccharide used to preserve embryo integrity. Constructs injected as mRNA were GFP-FAK (200pg), constitutively active Src Y527F (500pg), E-cadherin (800pg), N-cadherin (800pg), p120-catenin GFP (200pg), membrane RFP and GFP (300pg) nuclear RFP (300pg). Src-FRET was injected as DNA (200pg). N-cadherin morpholino (8ng), sequence 5′-GAAGGGCTCTTTCCGGCACATGGTG-3’ as described in (Theveneau et al., 2010). *Xenopus* cranial NC cells were dissected from the embryo at stage 18 and plated on fibronectin coated dishes or coverslips as described in (Alfandari et al., 2003, Milet and Monsoro-Burq, 2014). Briefly, the vitelline envelopes are removed from the embryo at stage 16 and at stage 18 the neural crest is dissected from the embryos. The neural crest is lateral to the neural tube and is located underneath the pigmented outer layer of the ectoderm. It can be identified as a greyish mass of cells and is easily separated from the white cephalic mesoderm beneath to yield a pure population of neural crest. For single cell experiments and CIL experiments NC explants were incubated in Ca^2+^ and Mg^2+^ free medium in order to encourage dissociation. The NC were cultured in Danilchick’s medium. The transgenic zebrafish lines sox10:egfp and sox10mGFPnRFP were used. Zebrafish were injected with FRNK at 2-cell stage.

#### Identifying Free Edge, Cell-Cell Contact and Dynamic Protrusions

The free edge of cells was identified by the absence of yolk platelets (Figure S1E), and the cell-cell contact was identified by the increase in signal intensity of membrane staining (Figure S1F). To visualise and measure the size of protrusions (Figures 1A and 1B; Figure S1B), extension analysis was carried out on cells expressing membrane RFP/GFP. The thresholded image from one time-point is subtracted from the thresholded image of the next time-point so the area of protrusion extension can be measured. This is then visualised by overlaying this region in red on the membrance RFP/GFP image (Figure S1G)

#### Immunostaining & In Situ Hybridisation

NC explants were plated on glass coverslips and left to spread. They were fixed in 3.7% formaldehyde for 30 minutes. They were permeabilised in 0.2% Triton X 100 and blocked in 2% serum. Primary antibodies used were phospho-paxillin pY118 (1:200; ThermoFisher), vinculin (1:200; Sigma) Src active antibody clone 28 (1:200; ThermoFisher), N-cadherin MNCD2 (1:100; DSHB) and E-cadherin (1:200; DSHB). Secondary antibodies were IgG Alexa based antibodies (1:250; Invitrogen). Counterstains used were Hoescht 3342 (1:1000; Thermofisher) and Phalloidin Rhodamine (1:500; Sigma). Cells were mounted in Mowiol and imaged on a Leica TCS SPE upright confocal microscope using a 63x oil immersion lens and a 1.5x digital zoom. Image analysis was carried out on ImageJ. All cells in the field of view were included in the analysis. To determine CMA area, images were thresholded and regions of interest drawn [as illustrated in Figure 2F]. Thresholding was constant for all conditions for a given experiment. For *in situ* hybridisation embryos were fixed at stage 24 and *in situ* hybridisation was carried out as described in (Harland, 1991); briefly, fixed embryos were incubated overnight with specific antisense RNA probes labelled with UTP-digoxigenin and a colorimetric reaction was used to develop the localization of an antibody against digoxigenin. The NC was labelled with a digoxigenin-labelled RNA *Twist* probe.

#### Single Cell Collisions and Dispersion Movies

Time lapse movies of single cell collisions and dispersion were carried out on upright compound microscopes with motorised stages, either a DM550B Leica Microscope with a DFC 300FX Leica camera or a DMRXA2 Leica Microscope with a Hamamatsu Digital camera. A 10x objective was used to image dispersion while a 20x objective was used to image single cell collisions. Dispersion was analysed using Delaunay triangulation, using the nuclear marker as the point for each cell as previously described (Carmona-Fontaine et al., 2011). This method measures the area of the triangle between each cell and its closest neighbours.

#### GFP-FAK Analysis

Live movies of GFP-FAK and membraneRFP were generated using a VoX spinning disk confocal microscope with a 60x lens or a Leica TCS SP8 with a 63x lens. The longevity and disassembly rates of CMAs were analysed using the Focal Adhesion Analysis Server (Berginski and Gomez, 2013).

#### Traction Force Microscopy

Traction force microscopy was performed as previously described (Theveneau et al., 2013); briefly, polyacrylamide hydrogels containing fluorescent beads were prepared at 600Pa and bead displacement was measured. Traction forces were calculated as previously described (Lin et al., 2010); briefly, equations from Lin et al. (2010) were implemented in Python to calculate traction forces from gel displacement and here reported as traction stress. Images were taken on an Axiovert 200M inverted Zeiss compound microscope with a motorised stage and using a 32x objective.

#### Inhibitors and Blocking Antibody

Src activity was inhibited by SU6656 inhibitor (Sigma) at 5μM for all *in vitro* assays and 100μΜ for inhibition of NC migration *in vivo*. FAK inhibitor PF-573228 (Sigma) was used to inhibit FAK activity at 2μM for all *in vitro* assays and 100μM for inhibition of NC migration *in vivo*. For the *in vivo* experiments, embryos were incubated in the inhibitors from stage 18 until stage 24. N-cadherin was inhibited with the blocking antibody-CDH2 (Life Technologies) by incubating the explants in the blocking antibody for 40 minutes at 100μg.ml^-1^ before they were plated. Contractility was inhibited with the ROCK inhibitor Y-27632 at 10μM.

#### Laser Ablation

Laser ablation experiments were carried out on an Olympus FV1000 microscope using a 60x lens. CMAs and CCAs were ablated for 30 seconds over an area of 50 μm^2^ using a PicoQuant picosecond pulsed diode laser turned to 405 nm at 2.3mW with 40 MHz repetition rate. Images were acquired every 30 seconds.

#### Cell-Cell Adhesion/ N-cadherin Strength Assays

For the cell-cell adhesion assay a monolayer of WT NC cells were plated out and allowed to attach. Cells labelled with Rhodamine Dextran (thermoFisher) were then plated on top of the monolayer and allowed to attach. An image was taken of the dish to show the amount of fluorescently labelled cells plated. The dish was then flipped and a second image was taken. A ratio was taken of the total area of fluorescence in the post-flipped image over the pre-flipped image to determine the percentage of cells that remained attached. For the N-cadherin strength assay, plastic dishes were incubated in 2μg.ml^-1^ of the N-cadherin Fc chimera protein (R&D systems) for 1 hour. Rhodamine Dextran labelled explants were then plated on the N-cadherin substrate and allowed to attach before the dish was flipped. Once again an image was acquired before and after flip to determine the percentage of explants that remained adhered.

### Quantification and Statistical Analysis

Statistical analyses were carried out using PRISMA. The types of statistical tests, exact value of n, what n represents, definition of center, and dispersion and precision measures are mentioned for each experiment in the corresponding figure legend. For significance we used the convention: ^∗∗∗^ = p≤0.001, ^∗∗^ = p≤0.01, ^∗^ = p≤0.05. The observed statistical effects were large therefore small sample sizes were sufficient to determine significance. D’Agostino’s K-squared test was used to assess normality of the data sets.

## Supplemental Information

Document S1. Figures S1–S5

Document S2. Article plus Supplemental Information

## References

[bib1] Abercrombie M. (1970). Contact inhibition in tissue culture. In Vitro.

[bib2] Abercrombie M., Ambrose E.J. (1958). Interference microscope studies of cell contacts in tissue culture. Exp. Cell Res..

[bib3] Abercrombie M., Dunn G.A. (1975). Adhesions of fibroblasts to substratum during contact inhibition observed by interference reflection microscopy. Exp. Cell Res..

[bib4] Abercrombie M., Heaysman J.E. (1953). Observations on the social behaviour of cells in tissue culture. I. Speed of movement of chick heart fibroblasts in relation to their mutual contacts. Exp. Cell Res..

[bib5] Abercrombie M., Heaysman J.E. (1954). Observations on the social behaviour of cells in tissue culture. II. Monolayering of fibroblasts. Exp. Cell Res..

[bib6] Abercrombie M., Heaysman J.E., Karthauser H.M. (1957). Social behaviour of cells in tissue culture. III. Mutual influence of sarcoma cells and fibroblasts. Exp. Cell Res..

[bib7] Alfandari D., Cousin H., Gaultier A., Hoffstrom B.G., DeSimone D.W. (2003). Integrin alpha5beta1 supports the migration of *Xenopus* cranial neural crest on fibronectin. Dev. Biol..

[bib8] Ananthakrishnan R., Ehrlicher A. (2007). The forces behind cell movement. Int. J. Biol. Sci..

[bib9] Anear E., Parish R.W. (2012). The effects of modifying RhoA and Rac1 activities on heterotypic contact inhibition of locomotion. FEBS Lett..

[bib10] Astin J.W., Batson J., Kadir S., Charlet J., Persad R.A., Gillatt D., Oxley J.D., Nobes C.D. (2010). Competition amongst Eph receptors regulates contact inhibition of locomotion and invasiveness in prostate cancer cells. Nat. Cell Biol..

[bib11] Bahm I., Barriga E.H., Frolov A., Theveneau E., Frankel P., Mayor R. (2017). PDGF controls contact inhibition of locomotion by regulating N-cadherin during neural crest migration. Development.

[bib12] Batson J., Astin J.W., Nobes C.D. (2013). Regulation of contact inhibition of locomotion by Eph-ephrin signalling. J. Microsc..

[bib13] Batson J., Maccarthy-Morrogh L., Archer A., Tanton H., Nobes C.D. (2014). EphA receptors regulate prostate cancer cell dissemination through Vav2-RhoA mediated cell-cell repulsion. Biol. Open.

[bib14] Becker S.F., Mayor R., Kashef J. (2013). Cadherin-11 mediates contact inhibition of locomotion during *Xenopus* neural crest cell migration. PLoS One.

[bib15] Berginski M.E., Gomez S.M. (2013). The focal adhesion analysis server: a web tool for analyzing focal adhesion dynamics. F1000Res.

[bib16] Blake R.A., Broome M.A., Liu X., Wu J., Gishizky M., Sun L., Courtneidge S.A. (2000). SU6656, a selective Src family kinase inhibitor, used to probe growth factor signaling. Mol. Cell. Biol..

[bib17] Burute M., Thery M. (2012). Spatial segregation between cell-cell and cell-matrix adhesions. Curr. Opin. Cell Biol..

[bib18] Cai D., Chen S.C., Prasad M., He L., Wang X., Choesmel-Cadamuro V., Sawyer J.K., Danuser G., Montell D.J. (2014). Mechanical feedback through E-cadherin promotes direction sensing during collective cell migration. Cell.

[bib19] Calalb M.B., Polte T.R., Hanks S.K. (1995). Tyrosine phosphorylation of focal adhesion kinase at sites in the catalytic domain regulates kinase activity: a role for Src family kinases. Mol. Cell. Biol..

[bib20] Calalb M.B., Zhang X., Polte T.R., Hanks S.K. (1996). Focal adhesion kinase tyrosine-861 is a major site of phosphorylation by Src. Biochem. Biophys. Res. Commun..

[bib21] Camand E., Peglion F., Osmani N., Sanson M., Etienne-Manneville S. (2012). N-cadherin expression level modulates integrin-mediated polarity and strongly impacts on the speed and directionality of glial cell migration. J. Cell Sci..

[bib22] Carmona-Fontaine C., Matthews H.K., Kuriyama S., Moreno M., Dunn G.A., Parsons M., Stern C.D., Mayor R. (2008). Contact inhibition of locomotion in vivo controls neural crest directional migration. Nature.

[bib23] Carmona-Fontaine C., Theveneau E., Tzekou A., Tada M., Woods M., Page K.M., Parsons M., Lambris J.D., Mayor R. (2011). Complement fragment C3a controls mutual cell attraction during collective cell migration. Dev. Cell.

[bib24] Carragher N.O., Westhoff M.A., Fincham V.J., Schaller M.D., Frame M.C. (2003). A novel role for FAK as a protease-targeting adaptor protein: regulation by p42 ERK and Src. Curr. Biol..

[bib25] Case L.B., Waterman C.M. (2015). Integration of actin dynamics and cell adhesion by a three-dimensional, mechanosensitive molecular clutch. Nat. Cell Biol..

[bib26] Chen B.H., Tzen J.T., Bresnick A.R., Chen H.C. (2002). Roles of Rho-associated kinase and myosin light chain kinase in morphological and migratory defects of focal adhesion kinase-null cells. J. Biol. Chem..

[bib27] Coburn L., Lopez H., Caldwell B.J., Moussa E., Yap C., Priya R., Noppe A., Roberts A.P., Lobaskin V., Yap A.S. (2016). Contact inhibition of locomotion and mechanical cross-talk between cell-cell and cell-substrate adhesion determine the pattern of junctional tension in epithelial cell aggregates. Mol. Biol. Cell.

[bib28] Collins C., Nelson W.J. (2015). Running with neighbors: coordinating cell migration and cell-cell adhesion. Curr. Opin. Cell Biol..

[bib29] Davis J.R., Huang C.Y., Zanet J., Harrison S., Rosten E., Cox S., Soong D.Y., Dunn G.A., Stramer B.M. (2012). Emergence of embryonic pattern through contact inhibition of locomotion. Development.

[bib30] Davis J.R., Luchici A., Mosis F., Thackery J., Salazar J.A., Mao Y., Dunn G.A., Betz T., Miodownik M., Stramer B.M. (2015). Inter-cellular forces orchestrate contact inhibition of locomotion. Cell.

[bib31] Desai R.A., Gopal S.B., Chen S., Chen C.S. (2013). Contact inhibition of locomotion probabilities drive solitary versus collective cell migration. J. R. Soc. Interface.

[bib32] Ezratty E.J., Partridge M.A., Gundersen G.G. (2005). Microtubule-induced focal adhesion disassembly is mediated by dynamin and focal adhesion kinase. Nat. Cell Biol..

[bib33] Fincham V.J., Frame M.C. (1998). The catalytic activity of Src is dispensable for translocation to focal adhesions but controls the turnover of these structures during cell motility. EMBO J..

[bib34] Grashoff C., Hoffman B.D., Brenner M.D., Zhou R., Parson M., Yang M.T., McLean M.A., Sligar S.G., Chen C.S., Ha T., Schwartz M.A. (2010). Measuring mechanical tension across vinculin reveals regulation of focal adhesion dynamics. Nature.

[bib35] Harland R.M. (1991). In situ hybridization: an improved whole-mount method for *Xenopus* embryos. Methods Cell Biol..

[bib36] Harris A. (1973). Location of cellular adhesions to solid substrata. Dev. Biol..

[bib37] Hatta K., Takeichi M. (1986). Expression of N-cadherin adhesion molecules associated with early morphogenetic events in chick development. Nature.

[bib38] Heaysman J.E., Pegrum S.M. (1973). Early contacts between fibroblasts. An ultrastructural study. Exp. Cell Res..

[bib39] Huttenlocher A., Lakonishok M., Kinder M., Wu S., Truong T., Knudsen K.A., Horwitz A.F. (1998). Integrin and cadherin synergy regulates contact inhibition of migration and motile activity. J. Cell Biol..

[bib40] Ilić D., Furuta Y., Kanazawa S., Takeda N., Sobue K., Nakatsuji N., Nomura S., Fujimoto J., Okada M., Yamamoto T. (1995). Reduced cell motility and enhanced focal adhesion contact formation in cells from FAK-deficient mice. Nature.

[bib41] Jülich D., Cobb G., Melo A.M., McMillen P., Lawton A.K., Mochrie S.G., Rhoades E., Holley S.A. (2015). Cross-scale integrin regulation organizes ECM and tissue topology. Dev. Cell.

[bib42] Kadir S., Astin J.W., Tahtamouni L., Martin P., Nobes C.D. (2011). Microtubule remodelling is required for the front-rear polarity switch during contact inhibition of locomotion. J. Cell Sci..

[bib43] Kashef J., Franz C.M. (2015). Quantitative methods for analyzing cell-cell adhesion in development. Dev. Biol..

[bib44] Kawakatsu H., Sakai T., Takagaki Y., Shinoda Y., Saito M., Owada M.K., Yano J. (1996). A new monoclonal antibody which selectively recognizes the active form of Src tyrosine kinase. J. Biol. Chem..

[bib45] Kuriyama S., Theveneau E., Benedetto A., Parsons M., Tanaka M., Charras G., Kabla A., Mayor R. (2014). In vivo collective cell migration requires an LPAR2-dependent increase in tissue fluidity. J. Cell Biol..

[bib46] Leonard M., Zhang L., Bleaken B.M., Menko A.S. (2013). Distinct roles for N-Cadherin linked c-Src and fyn kinases in lens development. Dev. Dyn..

[bib47] Lin Y.C., Tambe D.T., Park C.Y., Wasserman M.R., Trepat X., Krishnan R., Lenormand G., Fredberg J.J., Butler J.P. (2010). Mechanosensing of substrate thickness. Phys. Rev. E Stat. Nonlin. Soft Matter Phys..

[bib48] Lin B., Yin T., Wu Y.I., Inoue T., Levchenko A. (2015). Interplay between chemotaxis and contact inhibition of locomotion determines exploratory cell migration. Nat. Commun..

[bib49] Loeb L. (1921). Amoeligboid movement, tissue formation and consistency of protoplasm. Science.

[bib50] Maruthamuthu V., Sabass B., Schwarz U.S., Gardel M.L. (2011). Cell-ECM traction force modulates endogenous tension at cell-cell contacts. Proc. Natl. Acad. Sci. USA.

[bib51] Matthews H.K., Marchant L., Carmona-Fontaine C., Kuriyama S., Larraín J., Holt M.R., Parsons M., Mayor R. (2008). Directional migration of neural crest cells in vivo is regulated by Syndecan-4/Rac1 and non-canonical Wnt signaling/RhoA. Development.

[bib52] Mayor R., Carmona-Fontaine C. (2010). Keeping in touch with contact inhibition of locomotion. Trends Cell Biol..

[bib53] Mayor R., Etienne-Manneville S. (2016). The front and rear of collective cell migration. Nat. Rev. Mol. Cell Biol..

[bib54] McLachlan R.W., Kraemer A., Helwani F.M., Kovacs E.M., Yap A.S. (2007). E-cadherin adhesion activates c-Src signaling at cell-cell contacts. Mol. Biol. Cell.

[bib55] McMillen P., Holley S.A. (2015). Integration of cell-cell and cell-ECM adhesion in vertebrate morphogenesis. Curr. Opin. Cell Biol..

[bib56] Mertz A.F., Che Y., Banerjee S., Goldstein J.M., Rosowski K.A., Revilla S.F., Niessen C.M., Marchetti M.C., Dufresne E.R., Horsley V. (2013). Cadherin-based intercellular adhesions organize epithelial cell-matrix traction forces. Proc. Natl. Acad. Sci. USA.

[bib57] Milet C., Monsoro-Burq A.H. (2014). Dissection of *Xenopus laevis* neural crest for in vitro explant culture or in vivo transplantation. J. Vis. Exp..

[bib58] Moore R., Theveneau E., Pozzi S., Alexandre P., Richardson J., Merks A., Parsons M., Kashef J., Linker C., Mayor R. (2013). Par3 controls neural crest migration by promoting microtubule catastrophe during contact inhibition of locomotion. Development.

[bib59] Mui K.L., Chen C.S., Assoian R.K. (2016). The mechanical regulation of integrin-cadherin crosstalk organizes cells, signaling and forces. J. Cell Sci..

[bib60] Myers J.P., Gomez T.M. (2011). Focal adhesion kinase promotes integrin adhesion dynamics necessary for chemotropic turning of nerve growth cones. J. Neurosci..

[bib61] Nakamura N., Tanaka J., Sobue K. (1993). Rous sarcoma virus-transformed cells develop peculiar adhesive structures along the cell periphery. J. Cell Sci..

[bib62] Owen J.D., Ruest P.J., Fry D.W., Hanks S.K. (1999). Induced focal adhesion kinase (FAK) expression in FAK-null cells enhances cell spreading and migration requiring both auto- and activation loop phosphorylation sites and inhibits adhesion-dependent tyrosine phosphorylation of Pyk2. Mol. Cell. Biol..

[bib63] Roycroft A., Mayor R. (2015). Forcing contact inhibition of locomotion. Trends Cell Biol..

[bib64] Roycroft A., Mayor R. (2016). Molecular basis of contact inhibition of locomotion. Cell. Mol. Life Sci..

[bib65] Sandilands E., Cans C., Fincham V.J., Brunton V.G., Mellor H., Prendergast G.C., Norman J.C., Superti-Furga G., Frame M.C. (2004). RhoB and actin polymerization coordinate Src activation with endosome-mediated delivery to the membrane. Dev. Cell.

[bib66] Sastry S.K., Burridge K. (2000). Focal adhesions: a nexus for intracellular signaling and cytoskeletal dynamics. Exp. Cell Res..

[bib67] Scarpa E., Roycroft A., Theveneau E., Terriac E., Piel M., Mayor R. (2013). A novel method to study contact inhibition of locomotion using micropatterned substrates. Biol. Open.

[bib68] Scarpa E., Szabó A., Bibonne A., Theveneau E., Parsons M., Mayor R. (2015). Cadherin switch during EMT in neural crest cells leads to contact inhibition of locomotion via repolarization of forces. Dev. Cell.

[bib69] Shao K., Chen Z.Y., Gautam S., Deng N.H., Zhou Y., Wu X.Z. (2016). Posttranslational modification of E-cadherin by core fucosylation regulates Src activation and induces epithelial-mesenchymal transition-like process in lung cancer cells. Glycobiology.

[bib70] Slack-Davis J.K., Martin K.H., Tilghman R.W., Iwanicki M., Ung E.J., Autry C., Luzzio M.J., Cooper B., Kath J.C., Roberts W.G. (2007). Cellular characterization of a novel focal adhesion kinase inhibitor. J. Biol. Chem..

[bib71] Smeets B., Alert R., Pešek J., Pagonabarraga I., Ramon H., Vincent R. (2016). Emergent structures and dynamics of cell colonies by contact inhibition of locomotion. Proc. Natl. Acad. Sci. USA.

[bib72] Stramer B., Mayor R. (2017). Mechanisms and in vivo functions of contact inhibition of locomotion. Nat. Rev. Mol. Cell Biol..

[bib73] Stramer B., Moreira S., Millard T., Evans I., Huang C.Y., Sabet O., Milner M., Dunn G., Martin P., Wood W. (2010). Clasp-mediated microtubule bundling regulates persistent motility and contact repulsion in *Drosophila* macrophages in vivo. J. Cell Biol..

[bib74] Tanaka M., Kuriyama S., Aiba N. (2012). Nm23-H1 regulates contact inhibition of locomotion, which is affected by ephrin-B1. J. Cell Sci..

[bib75] Theveneau E., Marchant L., Kuriyama S., Gull M., Moepps B., Parsons M., Mayor R. (2010). Collective chemotaxis requires contact-dependent cell polarity. Dev. Cell.

[bib76] Theveneau E., Steventon B., Scarpa E., Garcia S., Trepat X., Streit A., Mayor R. (2013). Chase-and-run between adjacent cell populations promotes directional collective migration. Nat. Cell Biol..

[bib77] Timpson P., Jones G.E., Frame M.C., Brunton V.G. (2001). Coordination of cell polarization and migration by the Rho family GTPases requires Src tyrosine kinase activity. Curr. Biol..

[bib78] Truffi M., Dubreuil V., Liang X., Vacaresse N., Nigon F., Han S.P., Yap A.S., Gomez G.A., Sap J. (2014). RPTPα controls epithelial adherens junctions, linking E-cadherin engagement to c-Src-mediated phosphorylation of cortactin. J. Cell Sci..

[bib79] Tsukita S., Oishi K., Akiyama T., Yamanashi Y., Yamamoto T. (1991). Specific proto-oncogenic tyrosine kinases of src family are enriched in cell-to-cell adherens junctions where the level of tyrosine phosphorylation is elevated. J. Cell Biol..

[bib80] Veracini L., Grall D., Schaub S., Beghelli-de la Forest Divonne S., Etienne-Grimaldi M.C., Milano G., Bozec A., Babin E., Sudaka A., Thariat J. (2015). Elevated Src family kinase activity stabilizes E-cadherin-based junctions and collective movement of head and neck squamous cell carcinomas. Oncotarget.

[bib81] Villar-Cerviño V., Molano-Mazón M., Catchpole T., Valdeolmillos M., Henkemeyer M., Martínez L.M., Borrell V., Marín O. (2013). Contact repulsion controls the dispersion and final distribution of Cajal-Retzius cells. Neuron.

[bib82] Wang Y., Botvinick E.L., Zhao Y., Berns M.W., Usami S., Tsien R.Y., Chien S. (2005). Visualizing the mechanical activation of Src. Nature.

[bib83] Webb D.J., Donais K., Whitmore L.A., Thomas S.M., Turner C.E., Parsons J.T., Horwitz A.F. (2004). FAK-Src signalling through paxillin, ERK and MLCK regulates adhesion disassembly. Nat. Cell Biol..

[bib84] Weber G.F., Bjerke M.A., DeSimone D.W. (2011). Integrins and cadherins join forces to form adhesive networks. J. Cell Sci..

[bib85] Westhoff M.A., Serrels B., Fincham V.J., Frame M.C., Carragher N.O. (2004). SRC-mediated phosphorylation of focal adhesion kinase couples actin and adhesion dynamics to survival signaling. Mol. Cell. Biol..

[bib86] Wilson P.D. (2011). Apico-basal polarity in polycystic kidney disease epithelia. Biochim. Biophys. Acta.

[bib87] Woo S., Rowan D.J., Gomez T.M. (2009). Retinotopic mapping requires focal adhesion kinase-mediated regulation of growth cone adhesion. J. Neurosci..

[bib88] Xing Z., Chen H.C., Nowlen J.K., Taylor S.J., Shalloway D., Guan J.L. (1994). Direct interaction of v-Src with the focal adhesion kinase mediated by the Src SH2 domain. Mol. Biol. Cell.

[bib89] Zaidel-Bar R., Cohen M., Addadi L., Geiger B. (2004). Hierarchical assembly of cell-matrix adhesion complexes. Biochem. Soc. Trans..

[bib90] Zaidel-Bar R., Milo R., Kam Z., Geiger B. (2007). A paxillin tyrosine phosphorylation switch regulates the assembly and form of cell-matrix adhesions. J. Cell Sci..

[bib91] Zimmermann J., Camley B.A., Rappel W.J., Levine H. (2016). Contact inhibition of locomotion determines cell-cell and cell-substrate forces in tissues. Proc. Natl. Acad. Sci. USA.

