# Supplementary material for: Redistribution of Adhesive Forces through Src/FAK Drives Contact Inhibition of Locomotion in Neural Crest
Source: Dev Cell. 2018 Jun 4;45(5):565–579.e3. doi: 10.1016/j.devcel.2018.05.003 (PMC5988567; doi:10.1016/j.devcel.2018.05.003)
Supplement: Document S1. Figures S1–S5 [file mmc1.pdf]

Developmental Cell, Volume *45*

Supplemental Information

Redistribution of Adhesive Forces through Src/FAK Drives Contact Inhibition of Locomotion in Neural Crest

Alice Roycroft, András Szabó, Isabel Bahm, Liam Daly, Guillaume Charras, Maddy Parsons, and Roberto Mayor

Supplemental Information

Redistribution of adhesive forces through Src/FAK drives contact inhibition of locomotion in neural crest

Alice Roycroft, András Szabó, Isabel Bahm, Liam Daly, Guillaume Charras, Maddy Parsons and Roberto Mayor

Supplemental figures

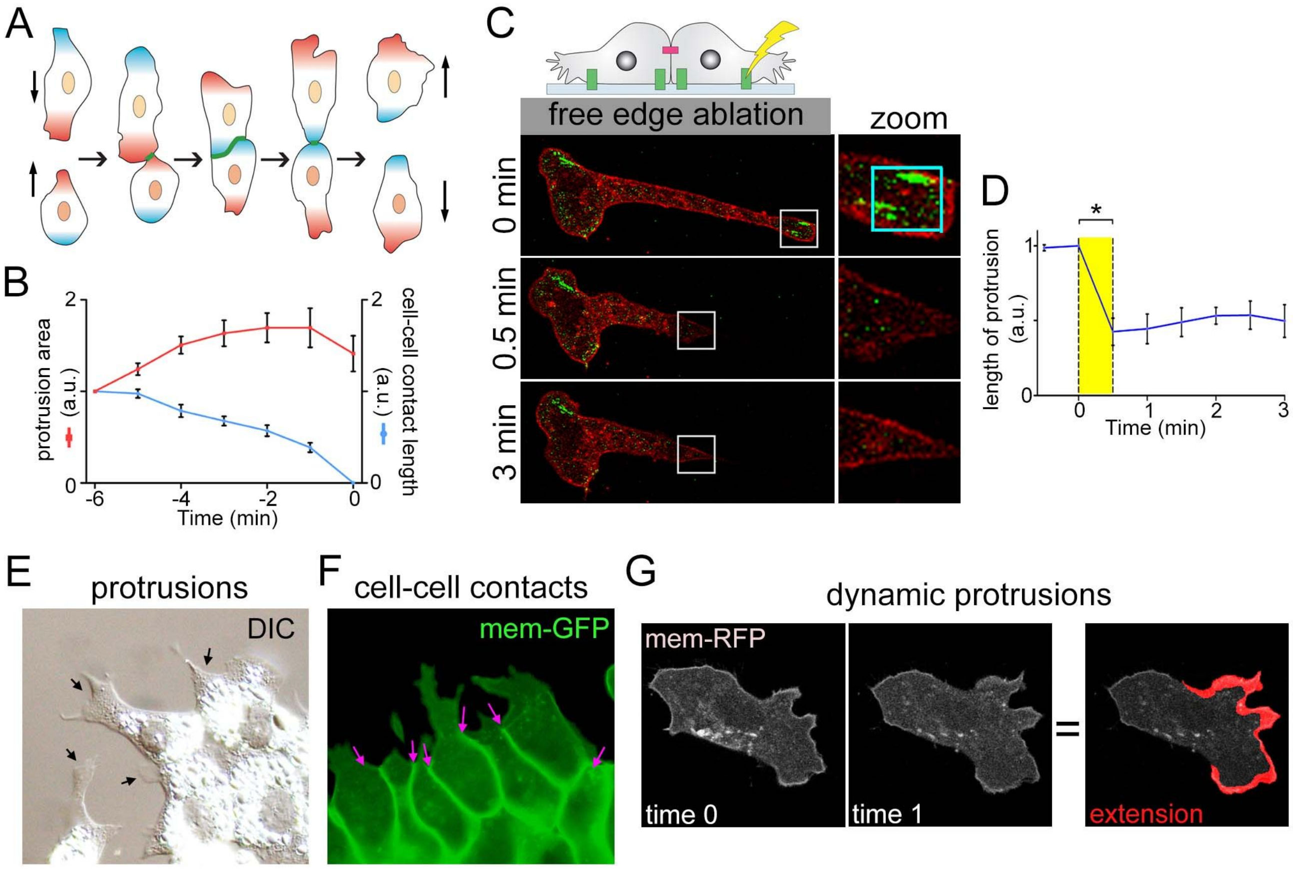

Figure S1. Stages of CIL. Related to Figure 1.
(A) Schematic illustration of CIL. Red: front (Rac activity), blue: rear (Rho activity), green: cell-cell contact. (B) Relative area of protrusion (red), as determined by extension subtraction analysis, and length of the cell-cell contact (blue) in control wild-type cells before separation (0 min). n = 14. (C) Schematic of cells and frames from movies of cell ablated at the cell-matrix adhesions in the free edge. Cells were injected to express membraneRFP (red) and GFP-FAK to label cell-matrix adhesions (green). Ablation area marked with a cyan box on zoom. (D) Length of protrusion after free edge ablation relative to length at the inititation of ablation (0 min). Yellow area indicates duration of laser ablation. n = 5. (E) Illustrative image of how the free edge of cells (arrow) was identified by the absence of birefringent yolk platelets (F) Illustrative image of how the cell-cell contact (arrow) was identified in cells expressing membrane GFP by the increase in membrane staining due to the overlapping of GFP signaling from adjacent cells. (G) Illustrative image of how protrusion area was visualised and measured by extension subtraction analysis. For cells expressing membrane RFP/GFP, one time-point is subtracted from the next time-point so the area of protrusion extension can be measured. This is then visualised by overlaying this region in red on the membrane RFP/GFP image. Line graph shows mean, errors ± SEM. * = p≤0.05

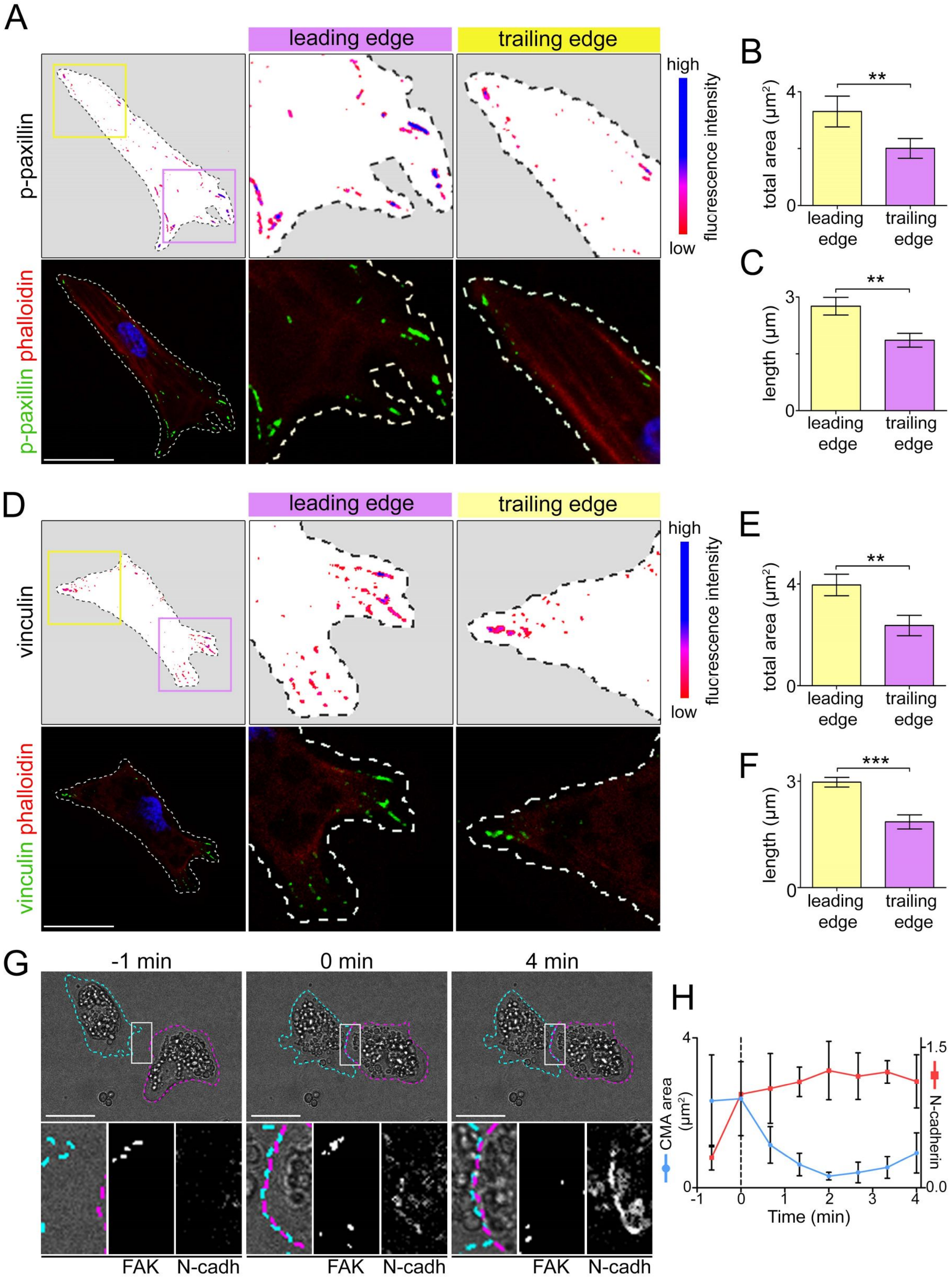

Figure S2. Cell-matrix adhesions reduced at rear of a single cell. Related to Figure 2.
(A, D) Immunocytochemistry on single cells against p-paxillin (A) or vinculin (D). Top: p-paxillin/vinculin coloured according to fluorescence intensity. Bottom: merged image of p-paxillin/vinculin (green), phalloidin (red) and Hoescht (blue). (B-C, E-F) Total area and length of p-paxillin/vinculin labelled CMAs. n = 23 cells. Boxes on images show region of zoom at leading edge (purple) and trailing edge (yellow). (G) Frames from a movie of two cells colliding labelled with N-cadherin-RFP and GFP-FAK, outlined in cyan and magenta on brightfield images. Box shows region of zoom at contact. (H) Total CMA area at contact upon a collision (blue) and N-cadherin recruitment to the contact quantified as fluorescence intensity (red). 0 min = first point of collision. n = 3 collisions. Scale bars 20 μm. Box graph show mean, errors ± SEM. *** = $p \leq 0.001$, ** = $p \leq 0.01$. Mann Whitney test.

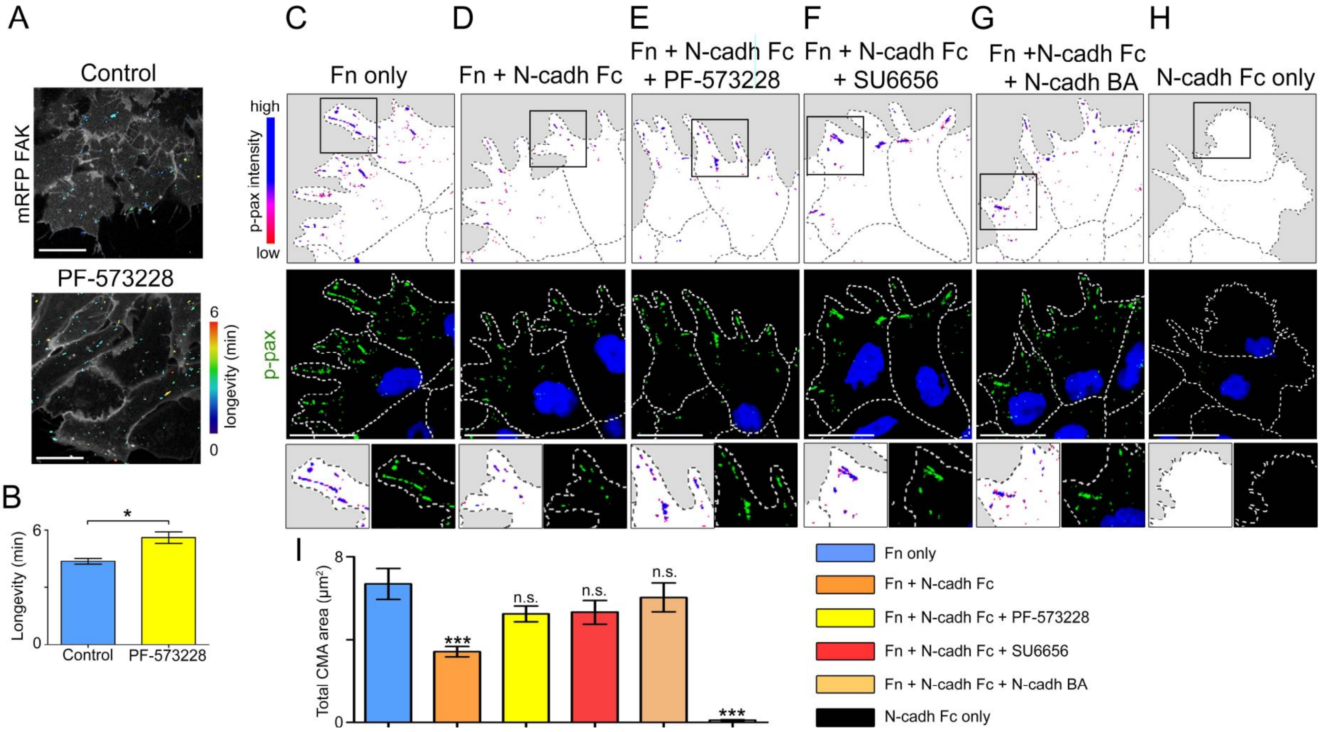

Figure S3. Loss of Src and FAK activity in stabilises CMAs. Related to Figure 5.
(A) Frame from movie of control cells or cells treated with PF-573228 (FAK inhibitor). Cells expressing membraneRFP (grey) and GFP-FAK (colour code indicates CMA longevity throughout movie). (B) Longevity of GFP-FAK positive CMAs near the contact. Control n = 29, PF-573228 n = 66. (C-H) Immunocytochemistry against p-paxillin coloured according to fluorescence intensity in cells plated on fibronectin at 10µg.ml^{-1} (C), cells plated on mixed substrate of fibronectin (10µg.ml^{-1}) and N-cadherin Fc (3µg.ml^{-1}) (D), cells incubated in PF-573228 FAK inhibitor and plated on fibronectin at 10µg.ml^{-1} with N-cadherin Fc at 3µg.ml^{-1} substrate (E), cells incubated in SU6656 Src inhibitor and plated on fibronectin at 10µg.ml^{-1} with N-cadherin Fc at 3µg.ml^{-1} substrate (F), cells pre-treated with N-cadherin BA and plated on fibronectin at 10µg.ml^{-1} with N-cadherin Fc at 3µg.ml^{-1} substrate (G), and cells plated on N-cadherin at 3µg.ml^{-1} only (H). P-paxillin (green), phalloidin (red) and Hoescht (blue) in merged image with cells outlined. Region of zoom shown in black boxes. (I) Total area of p-paxillin labelled CMAs per cell. Fn only n = 47, Fn + N-cadh Fc n = 48, Fn + N-cadh Fc + PF-573228 n = 29, Fn + N-cadh Fc + SU6656 n = 29, Fn + N-cadh Fc + N-cadh BA n = 35, N-cadh Fc only n = 14 cells. Scale bars 20µm. Bar graphs show mean, errors ± SEM. *** = p≤0.001, ** = p≤0.01, * = p≤0.05.

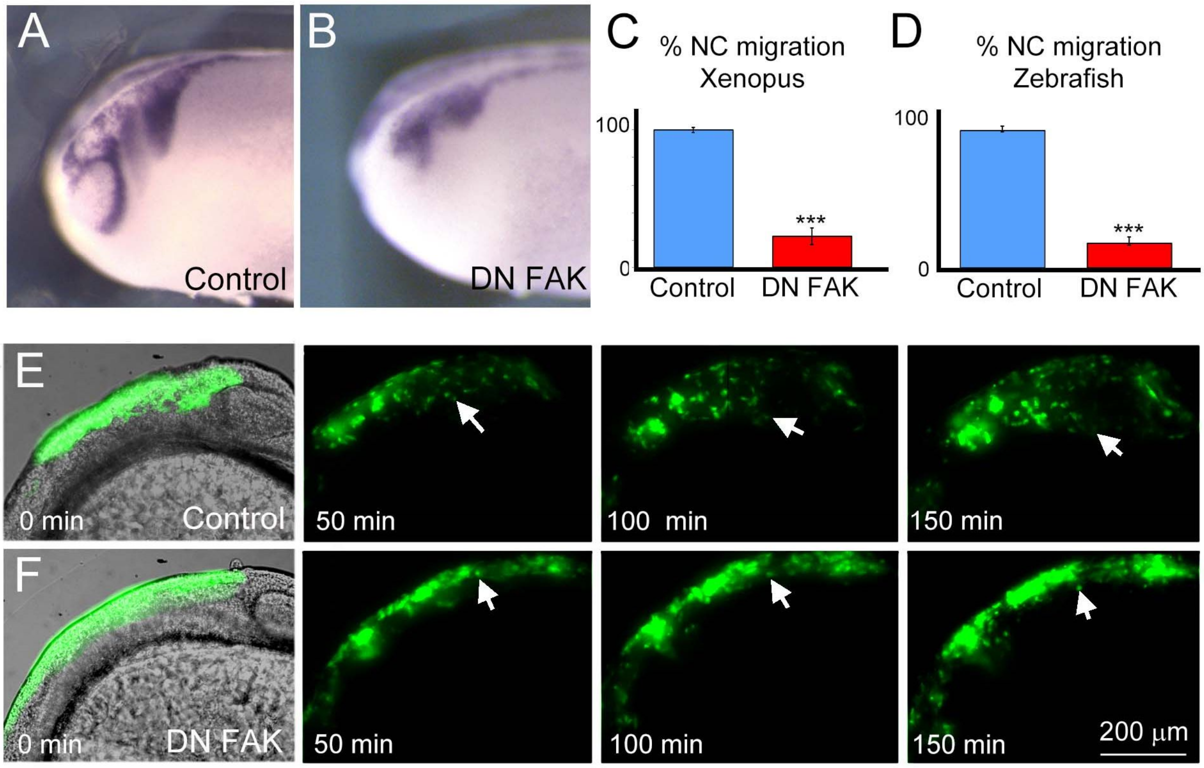

Figure S4. Inhibition of FAK reduces NC migration *in vivo*. Related to Figure 6.
(A,B) In situ hybridisation against *twist* to label the migrating NC in Xenopus laevis embryos. Control embryos (A) or embryos expressing FRNK – a dominant negative FAK (DN FAK) (B). (C,D) Quantification of the distance of migration where average control migration is 100%. 67 control and 55 DN FAJK embryos were anaylzed in the 3 independent experiments. (E,F) Frames from time-lapse movies of Sox10-GFP zebrafish embryos where the neural crest are expressing GFP for control embryos (E) and embryos expressing a dominant negative FAK (F). Bar graphs show mean, errors ± SEM. *** = $p \leq 0.001$. All ANOVA test.

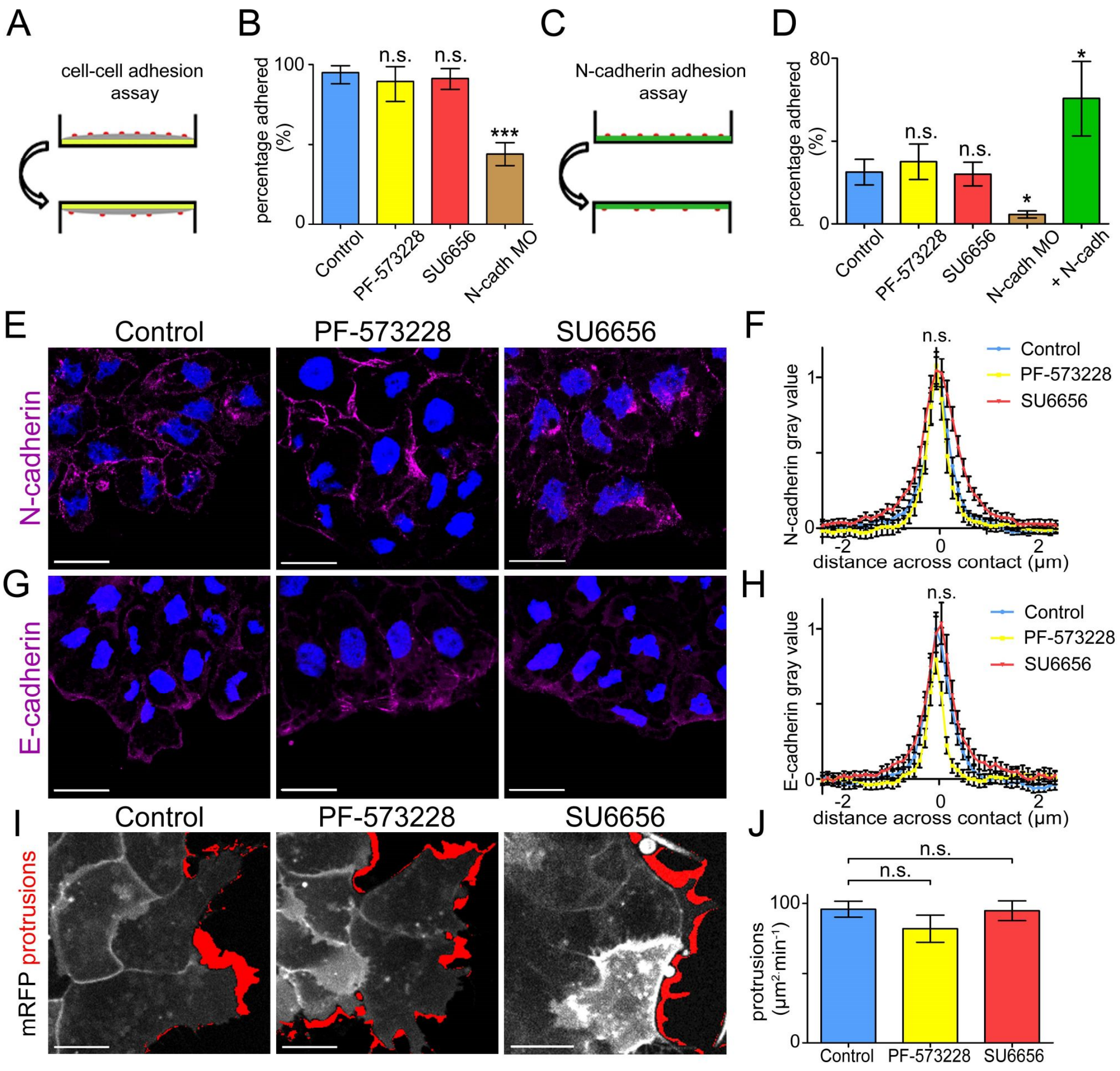

Figure S5: Src-FAK inhibition perturbs CIL without affecting cell-cell adhesions. Related to Figure 6.
(A) Schematic of cell-cell adhesion assay showing fibronectin (yellow), cell layer (grey) and fluorescently labelled cells (red). (B) Percentage of fluorescently injected cells that remained attached after flipping. n = 4 repeats. (C) Schematic of the N-cadherin adhesion assay showing N-cadherin substrate (green) and fluorescently labelled cells (red). (D) Percentage of explants that remain attached. n = 7 repeats for controls, PF-573228 and SU6656; n = 4 for N-cadherin MO injected cells, and n = 3 for cells overexpressing N-cadherin. (E) Immunocytochemistry against N-cadherin (magenta) and Hoescht (blue) in control cells, cells treated with PF-573228 (FAK inhibitor) and cells treated with SU6656 (Src inhibitor). (F) Fluorescence intensity of N-cadherin across cell-cell contacts with 0μm marking the centre of the contact. Values are normalised to average peak control levels for each repeat. The laser power was twice as high to image E-cadherin compared to N-cadherin. Control n = 52, PF-573228 n = 52, SU6656 n = 50 contacts. (G) Immunocytochemistry against E-cadherin (magenta) and Hoescht (blue) in control cells, cells treated with PF-573228 (FAK inhibitor) and cells treated SU6656 (Src inhibitor) (H) Fluorescence intensity of E-cadherin across contact with 0μm marking the centre of the contact. Values are normalised to average control levels in the cytoplasm for each repeat. Control n = 52, PF-573228 n = 39, SU6656 n = 29 contacts. (I) Protrusion extension analysis in control cells, cells treated with PF-573228 (FAK inhibitor) and cells treated with SU6656 (Src inhibitor), expressing membraneRFP (grey). Extension of protrusions in one minute is overlaid in red. (J) Extension area of protrusions per minute. Control n = 62, PF-573228 n = 30, SU6656U n = 31 cells. Scale bars 20μm. Line graphs and bar graphs show mean, errors ± SEM *** = p≤0.001, ** = p≤0.01, * = p≤0.05. ANOVA test.
